# Spatial analysis, coupling coordination, and efficiency evaluation of green innovation: A case study of the Yangtze River Economic Belt

**DOI:** 10.1371/journal.pone.0243459

**Published:** 2020-12-09

**Authors:** Ye Tian, Peng Huang, Xu Zhao

**Affiliations:** 1 College of Economics and Management, China Three Gorges University, Yichang, China; 2 Research Center for Reservoir Resettlement, China Three Gorges University, Yichang, China; Institute for Advanced Sustainability Studies, GERMANY

## Abstract

Green innovation is an important driving force to promote the sustainable development of urban society and economy. This paper constructs an evaluation index system containing social undesirable outputs, and uses the Super-SBM model and the Malmquist-Luenberger index to evaluate green innovation efficiency in 42 cities along the Yangtze River Economic Belt from 2013 to 2017. Additionally, spatial autocorrelation analysis is used to study the spatial correlation of green innovation efficiency. Finally, the coupling coordination degree model is used to study the coupling coordination degree between green innovation efficiency and high-tech industries. The following results were obtained. (1) The average value of green innovation efficiency increased from 1.0446 to 1.0987, and the annual average growth rate of total factor productivity of green innovation was 1.1%. (2) Green innovation efficiency of the Yangtze River Economic Belt had a significant spatial positive correlation, but the types of agglomeration among cities in different sections of the Yangtze River were quite different. (3) The coupling coordination degree between green innovation efficiency and the development level of high-tech industries in the cities of the Yangtze River Economic Belt was in the basic coordination stage. Based on the research results, we suggest that cities in this belt further enhance the interactive relationship between green innovation and economic development and promote the coordinated development of economy and society.

## Introduction

In the last few decades, countries have invested a large amount of resources to promote rapid economic development, but this has generated a large amount of pollution, and led to ecological destruction, resource depletion, and other problems on a global scale [1,2]. Especially in developing countries, the contradiction between economic development and environmental protection is becoming ever more prominent [3]. As one of the developing countries, China’s economy has developed rapidly since the reform and opening up. In 2019, the GDP per capita in China exceeded $10000, and a number of development regions with global influence have emerged, such as the Pearl River Delta city cluster, the Yangtze River Delta city cluster, and the Beijing-Tianjin-Hebei city cluster [4]. However, as a result of over-reliance on resources for development, many cities have overlooked their innovation ability and environmental problems, hindering their sustainable development in the process [5]. After 2012, China changed its focus to high-quality economic development instead of high-speed economic development. This transformation requires a reduction environment pollution and energy-intensive enterprises, strengthening of scientific and technological innovation, promotion of high-tech industries, and improvement in the quality of economic development [6,7]. In this light, green innovation and high-tech industries have become key factors for the promotion of regional economic transformation, while solving the problem of environmental pollution and achieving high-quality development. Accordingly, it is now important to study the efficiency and spatial agglomeration effect of green innovation, and the synergistic effect of green innovation and economic development on regional sustainable development.

Green innovation is generally defined as new or improved processes, technologies, systems, and products that avoid or reduce damage to the environment [8,9]. Green innovation is considered to be essential for social development and urban environmental transformation [10]. Green innovation efficiency is defined as the input-output efficiency of green innovation [11]. It is associated with various benefits, such as economic, environmental, social benefits, and has attracted widespread attention from scholars [12]. At present, existing research mainly focuses on the construction of an evaluation index system, selection of evaluation methods, and analysis of influencing factors.

With regard to construction of an evaluation index system, some scholars use a single technology index to measure green innovation efficiency [13]. However, a single indicator cannot truly reflect the scope of green innovation efficiency. This is why some scholars established an input-output evaluation system based on the Cobb-Douglas production function, Some scholars have established an input-output evaluation system based on Cobb-Douglas production function, by including R&D personnel full-time equivalent [14], government’s financial science and technology expenditure [15], enterprise’s internal science and technology expenditure [16], and energy consumption [11] as input indicators. With regard to the desirable output index, the existing research mainly considers the sales revenue of new products [9] and the number of invention patents [17] as good outputs. Some scholars have also added environmental benefits into the evaluation framework and added the environmental governance rate [18] into the index system as a comprehensive index. With regard to undesired outputs, existing studies mainly include the number of unauthorized patents [19], and environmental pollution indicators [12], such as sewage discharge, fixed wastes and air pollution into the evaluation system.

The evaluation methods for green innovation efficiency mainly include parametric methods (stochastic frontier analysis, or SFA) and non-parametric methods (data envelopment analysis, or DEA), because there is no need to set a production function and multiple output variables are possible. DEA and its improved model have become the mainstream choice for efficiency evaluation. Some scholars, such as Feng et al. [19], tend to use traditional DEA models to measure green innovation efficiency in China’s manufacturing industry. However, the traditional DEA model does not account for the impact of undesirable output on green innovation efficiency, and as a result, comparison is difficult when the number of effective decision-making units is large. Some scholars tend to use the Super-SBM model to calculate green innovation efficiency, as the results obtained with this model are comparable. For example, Long et al. [20] and Li et al. [21] used the Super-SBM model to measure the green innovation efficiency of each province in China, and Tang et al. [22] used the Super-SBM model to calculate the green innovation index of 496 A-share industrial enterprises in China.

After analyzing green innovation efficiency, most scholars, such as Zhang [15] and Yi [23], have carried out empirical analysis on the factors affecting the efficiency of green innovation. A few scholars have also studied the spatial agglomeration effect of green innovation efficiency. For example, Teng et al. [24] used spatial autocorrelation to analyze the spatial effect of green innovation efficiency in China, and Xu et al. [25] analyzed the space-time effect of inter-provincial green innovation efficiency in the Yangtze River Economic Belt by using spatial autocorrelation.

In summary, the existing research on green innovation efficiency mainly has the following gaps: (1) Most scholars have adopted the SBM-DEA model to measure green innovation efficiency. However, the maximum static efficiency measured by this model is 1, which does not accurately reflect the actual efficiency value of effective decision-making units. Moreover, when multiple effective decision-making units are included, the decision-making units cannot be compared effectively. A small number of scholars used Super-SBM to analyze the efficiency of green innovation, but they did not consider undesired outputs of a social nature. (2) Previous studies on spatial correlation were conducted in provincial regions, but the development of different cities in the same province may vary greatly. Therefore, the evaluation of green innovation efficiency in provincial regions cannot accurately and effectively reflect the development and efficiency of green innovation. (3) After evaluating green innovation efficiency, existing studies mostly use econometric models to analyze the factors that affect efficiency, and do not consider the suitability of the models to the economic development capabilities and resource utilization levels, or explore the constraints placed by high efficiency and low quantity on high-quality development. As a result, the general belief is that the better the economic development, the higher is the efficiency of green innovation. In order to fix these gaps, this paper firstly incorporated indexes of a social nature into the traditional index system, which only includes the economic and environmental index, to construct a more comprehensive evaluation index system. Next, in order to better distinguish the level of green innovation development in different cities within the same province, this paper uses the Super-SBM model to measure green innovation efficiency at the city level. Finally, this paper considers high-tech industries development as a contribution of green innovation at the economic level, and accordingly, constructs a coupling coordination degree model of green innovation efficiency and high-tech industries development to study the synergetic relationship between green innovation efficiency and economic development.

The main contributions of this paper are as follows. First, cities are used as research objects to explore green innovation efficiency from a more microscopic perspective, as a result of which accurate suggestions can be provided with regard to the development of green innovation in different cities. Second, the social nature of undesirable outputs is incorporated into the Super-SBM model in order to enrich the existing research index system. Third, the degree of interaction and influence between green innovation efficiency and economic development is quantitatively analyzed to incorporate the synergistic effect into the research framework and enrich the research perspective. Fourth, the analytical framework and methods used in this paper are universal in terms of index selection and research methods; therefore, the analytical framework used here would be relevant to research on green innovation in other countries too.

The rest of this paper is structured as follows: Section 2 introduces the study area and data. Section 3 illustrates the methods applied and construction of the models. Section 4 presents the results derived from the different models. Section 5 discusses the results and limitations. Section 6 presents the conclusions and offers recommendations.

## Study area and data

### Study area

**Fig 1:**
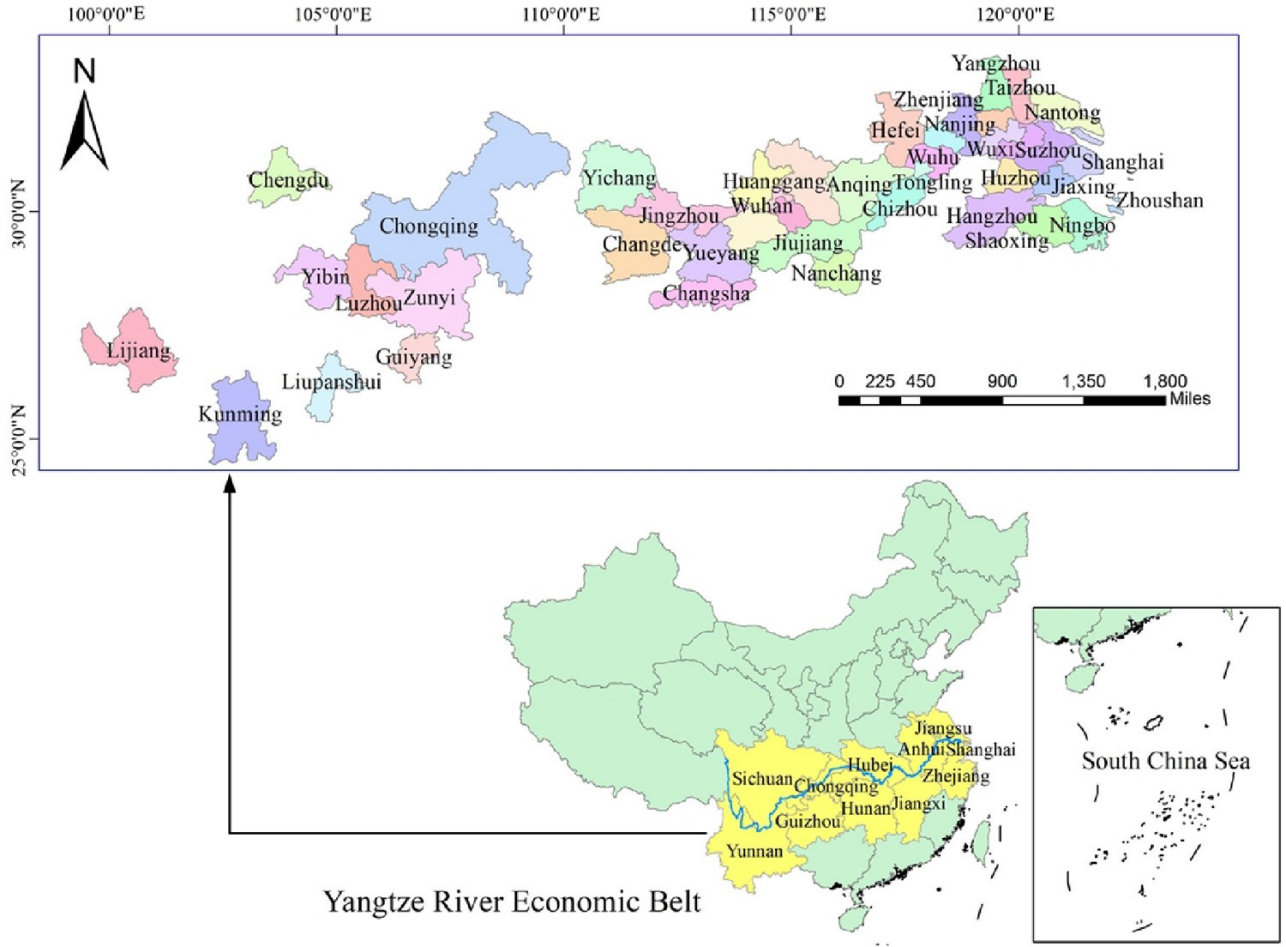
Research cities and locations for assessing green innovation efficiency.

The Yangtze River Economic Belt spans 11 provinces and municipalities across the east, middle, and west of China, and accounts for 45% of the country’s total economic output while covering 21% of the country’s land. It is not only a regional economic center for China’s new round of opening up and transformation, but also an inland river economic belt with global influence [26]. However the development of the regional economy has been accompanied by a series of environmental problems in the past. In 2018, the average R&D intensity of cities along the Yangtze River Economic Belt was only 90% of the national average, but its energy consumption and pollution intensity were more than double the national average [27]. This imbalance between controlling environmental pollution and achieving high-quality development has become a key factor restricting regional economic transformation. In 2016, China proposed that by 2020, in the Yangtze River Economic Belt, the ecological environment should be significantly improved, significant progress should be made in innovation-driven development, and the quality and efficiency of economic development should be significantly improved [28]. Another proposition was for the R&D investment intensity to increase to more than 2.5%, so that this region becomes a strategic support belt in leading national economic and social development [29]. In 2018, the state pointed out that it is necessary to give fully utilize the benefits of the geographical advantages of the Yangtze River Economic Belt, which straddles the eastern and western parts of the country, in order to strengthen scientific and technological innovation and promote high-quality development of this region [30]. Therefore, this article includes 38 major cities along the Yangtze River that are involved in the Development Plan Outline of the Yangtze River Economic Belt, as well as the provincial capital cities of the provinces along the Yangtze River. Finally, 42 cities were selected as the research object, and according to their location with respect to the river, they were divided into upstream, midstream, and downstream cities (Table 1) [31]. The research area is shown in Fig 1.

**Table 1:** Cities included as decision-making units.

River section	City
**Upstream**	Lijiang, Kunming, Liupanshui, Guiyang, Zunyi, Chengdu, Yibin, Luzhou, Chongqing
**Midstream**	Yichang, Jingzhou, Changde, Changsha, Yueyang, Xianning, Wuhan, Ezhou, Huanggang, Huangshi, Nanchang, Jiujiang
**Downstream**	Anqing, Chizhou, Tongling, Hefei, Wuhu, Maanshan, Nanjing, Zhenjiang, Yangzhou, Taizhou, Changzhou, Wuxi, Nantong, Hangzhou, Shaoxing, Huzhou, Jiaxing, Suzhou, Shanghai, Ningbo, Zhoushan

### Data collection

In space, 42 major cities in the Yangtze River Economic Belt were selected as the research object (Table 1). The Yangtze River Economic Belt came under the national limelight as a region of strategic importance in 2013. Therefore, in order to accurately measure the impact of strategic improvement in green innovation efficiency, the years from 2013 to 2017 are considered as the evaluation years. Based on the authoritativeness, availability, and continuity of the data, data on R&D personnel full-time equivalent, internal R&D expenditures, number of unauthorized patents, and number of invention patents granted were obtained from China City Statistical Yearbook (2014–2018) [26]; data on fiscal expenditure on science and technology, revenue from new product sales, “green governance” inflation pressure, and “transformative” unemployment rate were obtained from China Statistical Yearbook and China City Statistical Yearbook (2014–2018) [32]; data on per unit GDP energy consumption were from China Energy Statistics Yearbook (2014–2018) [33]. The comprehensive environmental treatment rate was calculated using the entropy method based on three indexes, namely, excellent air quality rate, comprehensive utilization rate of fixed wastes, and centralized treatment rate of waste water, which were all derived from China Environmental Statistics Yearbook (2014–2018) [33]. Interpolation was used to make up for missing data from any of the years. Descriptive statistics and data sources of each variable are shown in Table 2.

**Table 2:** Descriptive statistics and data sources.

Variable	Mean	Std.Dev.	Min	Max	Data sources
R&D personnel full-time equivalent	26550.14	36376.01	872	184000	China City Statistical Yearbook
Internal R&D expenditures	1060000	1700000	15771	12100000	China City Statistical Yearbook
Fiscal expenditure on science and technology	271000	499000	12130	3900000	China Statistical Yearbook
Per unit GDP energy consumption	0.567	0.37	0.116	1.729	China Energy Statistics Yearbook
Number of unauthorized patents	12162.26	14443.16	114	72868	China City Statistical Yearbook
“Green governance” inflation pressure	0.019	0.006	0.002	0.039	China Statistical Yearbook
“Transformative” unemployment rate	0.028	0.008	0.013	0.045	China Statistical Yearbook
Number of invention patents granted	2204.538	3417.924	10	20681	China City Statistical Yearbook
Revenue from new product sales	14300000	18500000	57821	101000000	China Statistical Yearbook
Comprehensive environmental treatment rate	0.819	0.071	0.507	0.958	China Environmental Statistics Yearbook

## Methods

In this paper, in order to get a clear grasp of the development level of green innovation in the Yangtze River Economic Belt, the Super-SBM model was used to measure the static efficiency of green innovation. Second, the Malmquist-Luenberger index was used to analyze the dynamic efficiency of green innovation in the Yangtze River Economic Belt and its driving factors from 2013 to 2017. Following this, spatial autocorrelation analysis was used to analyze the spatial distribution of green innovation efficiency. Finally, the coupling coordination degree model was used to quantitatively analyze the interaction and degree of influence between green innovation efficiency and economic development.

### Construction of an evaluation index system

Based on actual data on the development of the Yangtze River Economic Belt from the technological, ecological, and environmental perspectives and the green innovation efficiency evaluation index system constructed in the previous literature, this paper incorporates social indicators into the evaluation system and constructs an index system to measure green innovation efficiency from the economy, ecology, and society perspectives. The index system is shown in Table 3.

**Table 3:** Evaluation index system for green innovation efficiency.

Indicator type	First-level indicators	Secondary indicators	References
**Input indicators**	Human input	R&D personnel full-time equivalent	Chen [14]
	Resources input	Per unit GDP energy consumption	Liu [17]
	Capital inputs	Fiscal expenditure on science and technology	Zhang [15]; Clausen [34]
		Internal R&D expenditures	Sueyoshi [16]
**Output indicators**	Technological benefit	Number of invention patents granted	Liu [17]; Cuerva [35]
	Economic benefit	Revenue from new product sales	Liu [9]; Cuerva [35]
	Environmental benefit	Comprehensive environmental treatment rate	Du [18]; Song [36]
	Undesirable outputs	Number of unauthorized patents	Feng [19]; Carayannis [37]
		“Green governance” inflation pressure	LYU [38]
		“Transformative” unemployment rate	Reiff [39]

With regard to the input indicators, according to the Cobb-Douglas production function [40], research on green innovation efficiency generally uses three kinds of input: capital, human and resources [41]. The existing literature mostly considers R&D personnel full-time equivalent [14], and per unit GDP energy consumption [15] as human input and resources input, respectively, and internal R&D expenditures as capital input [42]. Government support is an important factor that affects the innovation ability of cities [34]. Therefore, this article adds fiscal expenditure on science and technology as innovation capital input.

With regard to desirable output indicators, green innovation can promote economic, technological, and environmental development, so most scholars choose the number of invention patents granted [9] and revenue from new product sales [35] as the output of technological benefits and economic benefits. Green innovation is a comprehensive consideration of energy conservation and a measure to improve the innovative development of the ecological environment [36]. Here, we used the comprehensive environmental governance rate to characterize environmental benefits. The cities in the Yangtze River Economic Belt focus on water, atmosphere, and fixed waste as aspects of comprehensive environmental governance. In this respect, the excellent air quality rate, the comprehensive utilization rate of fixed waste, and the centralized treatment rate of wastewater are used to determine the comprehensive environmental treatment rate [43].

With regard to undesirable output, not all patents filed are granted, and the number of unauthorized patents related to green innovation cannot bring actual benefits [37] and may lead to a waste of resources. This may be an indicator of undesirable output from the technology aspect. The input of green innovation forces the urban industry to transform from labor-intensive to technology-intensive and capital-intensive industries. This will lead to the creation of a large number of new jobs, but the transition from low-end industries to high-end industries will be accompanied by structural unemployment and “transitional” unemployment, which is one of the socially undesirable outputs [39]. At the same time, increasing the city’s investment in green innovation will cause large-scale agglomeration of production factors, subject to the constraints of the external environment. Accordingly, urban price levels will continue to rise and even cause inflation; such “green governance” inflation stress is also considered as an undesirable output [38].

### Super-SBM model

Among various methods used for objective weighting, factor analysis and principal component analysis are relatively simple. SFA is a parametric analysis method that is very effective in determining the efficiency when there are several input indicators and only one output index [13]. However, the characteristics of the input and output indicators need to be set in advance according to the specific function form. In contrast, DEA is a nonparametric analysis method that can solve the problem of multiple input and multiple output indicators without the production function being set [19]. As the efficiency of green innovation is dependent on multiple input and multiple output factors, we chose DEA for evaluating green innovation efficiency. The DEA model was proposed by Charnes et al. for the evaluation of relative efficiency of multiple decision-making units with multiple inputs and multiple outputs [44]. Since the traditional DEA model does not consider the correlation between the input-output indicators, it is not suitable for output indicators with negative externalities such as environmental pollution. Thus, Tone [45] incorporated the undesirable output into the DEA model and proposed a non-radial SBM-DEA model based on measured relaxation variables. Although the SBM-DEA model can avoid errors caused by relaxation variables and angle selection, the maximum efficiency value of this model is 1. However, if multiple cities become optimal decision-making units, effective comparison between cities cannot be carried out. In order to fix the shortcomings of the SBM model, this paper uses the Super-SBM model that considers slack variables [46], which allow the green innovation efficiency of city k to be higher than 1. The Super-SBM model can be used to effectively compare decision-making units with an efficiency value of more than 1, as shown below.

$$\rho^{*}=\text{min}\frac{\frac{1}{m}\sum_{i=1}^{m}\frac{{\bar{x}}_{i}}{x_{i0}}}{\frac{1}{s_{1}+s_{2}}\left(\sum_{r=1}^{s_{1}}\frac{{\bar{y}}_{r}^{g}}{y_{r0}^{g}}+\sum_{r=1}^{s_{2}}\frac{{\bar{y}}_{r}^{b}}{y_{r0}^{b}}\right)}$$

$$\text{s}.\text{t}.\{\begin{array}{c} \bar{x}\geq \sum_{j=1,\neq 0}^{n}\lambda_{j}x_{ij}\\ {\bar{y}}^{g}\leq \sum_{j=1,\neq 0}^{n}\lambda_{j}y_{rj}^{g}\\ {\bar{y}}^{b}\geq \sum_{j=1,\neq 0}^{n}\lambda_{j}y_{rj}^{b}\\ \bar{x}\geq x_{0},\;{\bar{y}}^{g}\leq y_{0}^{g},\;{\bar{y}}^{b}\geq y_{0}^{b},\;{\bar{y}}^{g}\geq 0,\;\lambda \geq 0 \end{array}$$ (1)

In the equation, ρ* is the value of green innovation efficiency; *n* is the number of samples; *x*, *y*^*g*^, and *y*^*b*^ represent input, desirable output and undesirable output, respectively; *m* indicates the number of input indicators; *s*_1_ indicates the desirable output indicators; *s*_2_ indicates undesirable output indicators; and *λ*_*j*_ is the weight vector. When ρ* ≥ 1, the decision-making unit is relatively effective; when ρ* < 1, the decision-making unit is relatively invalid.

### Malmquist-Luenberger index

After determining the static green innovation efficiency through the Super-SBM model, this paper uses the Malmquist-Luenberger index (ML index), which includes undesirable outputs to study the dynamic changes in green innovation efficiency and its driving factors [47]. The direction vector is *g*^*t*^ = *y*^*t*^ − *z*^*t*^ and the ML index from period *t* to *t* + 1 is as follows.

$$ML_{t}^{t+1}={\left[\frac{1+{\vec{D}}_{0}^{t}\left(x^{t},y^{t},z^{t};y^{t},-z^{t}\right)}{1+{\vec{D}}_{0}^{t}\left(x^{t+1},y^{t+1},z^{t+1};y^{t+1},-z^{t+1}\right)}\times \frac{1+{\vec{D}}_{0}^{t+1}\left(x^{t},y^{t},z^{t};y^{t},-z^{t}\right)}{1+{\vec{D}}_{0}^{t+1}\left(x^{t+1},y^{t+1},z^{t+1};y^{t+1},-z^{t+1}\right)}\right]}^{\frac{1}{2}}$$ (2)

*x*, *y*, and *z* represent the input, expected output, and undesirable output, respectively, of the decision-making unit; ${\vec{D}}_{0}^{t}\left(x^{t},y^{t},z^{t};y^{t},-z^{t}\right)$ is the direct distance function of the *t* period of the decision-making unit; ${\vec{D}}_{0}^{t+1}\left(x^{t+1},y^{t+1},z^{t+1};y^{t+1},-z^{t+1}\right)$ is the direct distance function of the *t* + 1 period; and ${ML}_{t}^{t+1}$ is the total factor productivity of the change index from period t to *t* + 1. When ${ML}_{t}^{t+1}>1$, it means that the green innovation total factor productivity has improved. When ${ML}_{t}^{t+1}<1$, it indicates that the green innovation total factor productivity has deteriorated. When ${ML}_{t}^{t+1}=1$, it indicates that the green innovation total factor productivity has remained unchanged. The ML index can be decomposed into technology efficiency change (EFFCH) and technical change (TECH), which can be expressed as follows.

$$ML_{t}^{t+1}=EFFCH_{t}^{t+1}\times TECH_{t}^{t+1}$$ (3)

$$TECH_{t}^{t+1}={\left[\frac{1+{\vec{D}}_{0}^{t+1}\left(x^{t},y^{t},z^{t};y^{t},-z^{t}\right)}{1+{\vec{D}}_{0}^{t}\left(x^{t+1},y^{t+1},z^{t+1};y^{t+1},-z^{t+1}\right)}\times \frac{1+{\vec{D}}_{0}^{t+1}\left(x^{t},y^{t},z^{t};y^{t},-z^{t}\right)}{1+{\vec{D}}_{0}^{t}\left(x^{t+1},y^{t+1},z^{t+1};y^{t+1},-z^{t+1}\right)}\right]}^{\frac{1}{2}}$$ (4)

$$EFFCH_{t}^{t+1}=\frac{1+{\vec{D}}_{0}^{t}\left(x^{t},y^{t},z^{t};y^{t},-z^{t}\right)}{1+{\vec{D}}_{0}^{t}\left(x^{t+1},y^{t+1},z^{t+1};y^{t+1},-z^{t+1}\right)}$$ (5)

In the equation, ${TECH}_{t}^{t+1}$ and ${EFFCH}_{t}^{t+1}$ represent the change index of technical change and the technology efficiency change, respectively, from period *t* to *t* + 1. When ${TECH}_{t}^{t+1}$ and ${EFFCH}_{t}^{t+1}$ are greater than 1, it indicates improvement in technical change and technology efficiency change. When ${TECH}_{t}^{t+1}$ and ${EFFCH}_{t}^{t+1}$ are less than 1, it indicates a reduction in technical change and technology efficiency change. When ${TECH}_{t}^{t+1}$ and ${EFFCH}_{t}^{t+1}$ are equal to 1, it indicates that technical change and technology efficiency change are unchanged.

### Spatial autocorrelation analysis

Spatial autocorrelation analysis is used to determine potential interdependencies between the observed data of some variables within the same distribution area, and includes global spatial autocorrelation and local spatial autocorrelation [48]. In this paper, global spatial autocorrelation and local spatial autocorrelation analysis are used to explore the spatial correlation and spatial variation of green innovation efficiency in the Yangtze River Economic Belt.

#### Spatial weight matrix

Before spatial autocorrelation analysis is performed, the spatial weight matrix of the study area must be set. This paper constructs a comprehensive economic-geographic distance weight matrix [49], as shown below.

$$W_{ij}=\{\begin{array}{c} \frac{1}{d_{ij}\times \left|{\bar{Y}}_{i}-{\bar{Y}}_{j}\right|}\left(i\neq j\right)\\ 0\left(i=j\right) \end{array}$$ (6)

*d*_*ij*_ is the spatial distance between the research objects *i* and *j*, and ${\overset{-}{Y}}_{i}$ and ${\overset{-}{Y}}_{j}$ are the average GDP of research objects *i* and *j*, respectively, during the research period.

#### Global space autocorrelation

Global spatial autocorrelation represents the overall spatial correlation of or spatial differences within the study area. This paper uses the global Moran’s I index to measure the overall spatial correlation of the green innovation efficiency of 42 cities in the Yangtze River Economic Belt. The equation is as follows.

$$I=\frac{\sum_{i=1}^{n}\sum_{j=1}^{n}W_{ij}\left(X_{i}-\bar{X}\right)\left(X_{j}-\bar{X}\right)}{S^{2}\sum_{i=1}^{n}\sum_{j=1}^{n}W_{ij}}$$ (7)

*n* is the number of study areas; *W*_*ij*_ is the spatial weight; *x*_*i*_ and *x*_*j*_ are the green innovation efficiency values of cities *i* and *j*, respectively; $\overset{-}{x}=\frac{1}{n}\sum_{i=1}^{n}x_{i}$ is the mean value of the green innovation efficiency of the study city; *S*^2^ is the observed value variance; and *I* is the global Moran’s I index. The value of the index ranges from -1 to 1. The closer the *I* value is to 1, the stronger the spatial agglomeration of the research object. The closer the *I* value is to -1, the stronger the spatial difference. *I* values close to 0 indicate that the research object does not have spatial correlation. The significance of Moran’s I index is determined using the Z statistic, the formula for which is as follows.

$$Z=\frac{Moran^{\prime }sI-E\left(Moran^{\prime }sI\right)}{\sqrt{VAR\left(Moran^{\prime }sI\right)}}$$ (8)

In the equation, *E*(*Moran’s I*) and *VAR*(*Moran’s I*) are the expected value and variance, respectively, of Moran’s I index. When the Z statistic is greater than 1.96, it indicates that Moran’s I index has significant spatial autocorrelation.

#### Local spatial autocorrelation

Local spatial autocorrelation indicates the spatial correlation or spatial heterogeneity of a certain attribute of an individual region in relation to its surrounding individual units. Usually, the local indices of spatial association (LISA) index is used to measure the degree of correlation and significance [50]. The common LISA analysis tools include local Moran’s I and Moran scatter plots. The equation is as follows.

$$I_{i}=\frac{\left(X_{i}-\bar{X}\right)}{S^{2}}\sum_{i\neq j}W_{ij}\left(X_{j}-\bar{X}\right)$$ (9)

When *I*_*i*_ is greater than 0, it indicates that the observed values of the research individuals are spatially high or low or concentrated; that is, a spatial agglomeration phenomenon is observed. When *I*_*i*_ is less than 0, it indicates that the observed values of the research individuals are spatially high or low or are highly or lowly concentrated, that is indicative of spatial heterogeneity. When *I*_*i*_ is equal to 0, it indicates that there is no spatial correlation between the study subjects.

The Moran scatter plot is an intuitive display of the local Moran’s I index of each research object in the quadrant graph. The horizontal and vertical axes of the quadrant graph represent the observations and spatial lag terms of the research object, respectively. The first and third quadrants represent positive spatial correlation: the former represents high-valued regions surrounded by high-valued regions (HH), and the latter represents low-valued regions surrounded by low-valued regions (LL). The second and fourth quadrants represent negative spatial correlations: the former represents low-value regions surrounded by high-value regions (LH), and the latter represents high-value regions are surrounded by low-value regions (HL).

### Coupling coordination degree

The coupling degree indicates the physical degree of interdependence between two or more entities [51], When applied to the field of social economics, it reflects the interaction and closeness between two or more social economic systems [52]. The coupling coordination degree can also be used as a measure of the degree of benign coupling [53]. The major cities in the Yangtze River Economic Belt have vigorously promoted the development of green innovation. However, with regard to the interaction between green innovation efficiency and economic development, it is not clear whether a higher level of urban economic development indicates higher efficiency of green innovation or whether improvement in green innovation efficiency promotes economic development. Few scholars have tried to explain or study these relationships. Therefore, this study uses the coupling coordination degree model to quantitatively analyze the interaction and degree of influence between green innovation efficiency and economic development. Firstly, in order to eliminate data dimensions and avoid the zero value of standardized data, an intercept term is added in the standardization [54,55], ass shown below.

For positive indicators:

$$X_{ijt}=\frac{x_{ijt}-minx_{jt}}{maxx_{jt}-minx_{jt}}\times 0.9+0.1$$ (10)

For negative indicators:

$$X_{ijt}=\frac{maxx_{jt}-x_{ijt}}{maxx_{jt}-minx_{jt}}\times 0.9+0.1$$ (11)

Where *x*_*ijt*_ indicates the original value of the *i* − *th* city for the *j* − *th* index in *t*; *max x*_*jt*_ and *min x*_*jt*_ indicate the maximum and minimum values, respectively, of the *j* − *th* index in *t*; *X*_*ijt*_ is the value after the variable is standardized.

Next, a coupling coordination model was constructed to examine the orderliness and overall coordination of the interaction between green innovation efficiency and the development level of high-tech industries in 42 cities in the Yangtze River Economic Belt. The model is shown below.

$$C=2{\left\{\frac{G_{1}\times E_{1}}{{\left(G_{1}+E_{1}\right)}^{2}}\right\}}^{\frac{1}{2}}$$ (12)

$$T=\alpha G_{1}+\beta E_{1}$$ (13)

$$D={\left(C\times T\right)}^{\frac{1}{2}}$$ (14)

In Eqs (12) to (14), *G*_1_ and *E*_1_ represent green innovation efficiency and the level of high-tech industries development, respectively; *C* represents the coupling degree; *T* represents the comprehensive reconciliation index of green innovation efficiency and the level of high-tech industries development; *D* represents the coordination degree; *α*, and *β* are the coefficients to be determined; and *α* + *β* = 1 mainly indicates the importance of each system [56]. In this paper, it is presumed that *α* = *β* = 0.5 [57]. According to the coupling coordination degree *D* and the measured values of *G*_1_ and *E*_1_, the cities can be classified as described by Cui [58] and Wu [59] (Table 4).

**Table 4:** Classification of coupling coordination degree.

Section	Classification	Calculation results	Characteristic
0 ≤ D < 0.2	No coordination	G_1_ –*E*_1_ > 0.1	High-tech industries development is blocked
		*E*_1_ –*G*_1_ > 0.1	Green innovation efficiency is blocked
		0 ≤ |*G*_1_−*E*_1_| ≤ 0.1	No coordination
0.2 ≤ D < 0.4	Low coordination	G_1_ –*E*_1_ > 0.1	High-tech industries development is blocked
		*E*_1_ –*G*_1_ > 0.1	Green innovation efficiency is blocked
		0 ≤ |*G*_1_−*E*_1_| ≤ 0.1	Low coordination
0.4 ≤ D < 0.6	Basic coordination	G_1_ –*E*_1_ > 0.1	High-tech industries development is blocked
		*E*_1_ –*G*_1_ > 0.1	Green innovation efficiency is blocked
		0 ≤ |*G*_1_−*E*_1_| ≤ 0.1	Basic coordination
0.6 ≤ D < 0.8	Good coordination	G_1_ –*E*_1_ > 0.1	High-tech industries development is blocked
		*E*_1_ –*G*_1_ > 0.1	Green innovation efficiency is blocked
		0 ≤ |*G*_1_−*E*_1_| ≤ 0.1	Good coordination
0.8 ≤ D < 1	Excellent coordination	G_1_ –*E*_1_ > 0.1	High-tech industries development is blocked
		*E*_1_ –*G*_1_ > 0.1	Green innovation efficiency is blocked
		0 ≤ |*G*_1_−*E*_1_| ≤ 0.1	Excellent coordination

## Results

### Static green innovation efficiency

The green innovation efficiency values of 42 major cities in the Yangtze River Economic Belt from 2013 to 2017 were calculated using MaxDEA 8 Pro, as shown in Table 4. From a global perspective, the average green innovation efficiency of the Yangtze River Economic Belt from 2013 to 2017 is 1.0694, and the overall input and output are in a valid stage. This indicates that the green innovation efficiency of the Yangtze River Economic Belt has been increasing on a yearly basis since 2013. From the perspective of time, the green innovation efficiency of major cities in the Yangtze River Economic Belt has shown an increasing trend: from 2013 to 2015, the green innovation efficiency increased gradually from 1.0446 to 1.0962. In 2016, the green innovation efficiency was significantly reduced to 1.0406, which is the lowest value in the past five years. In 2017, green innovation efficiency in the Yangtze River Economic Belt increased significantly to 1.0987.

**Fig 2:**
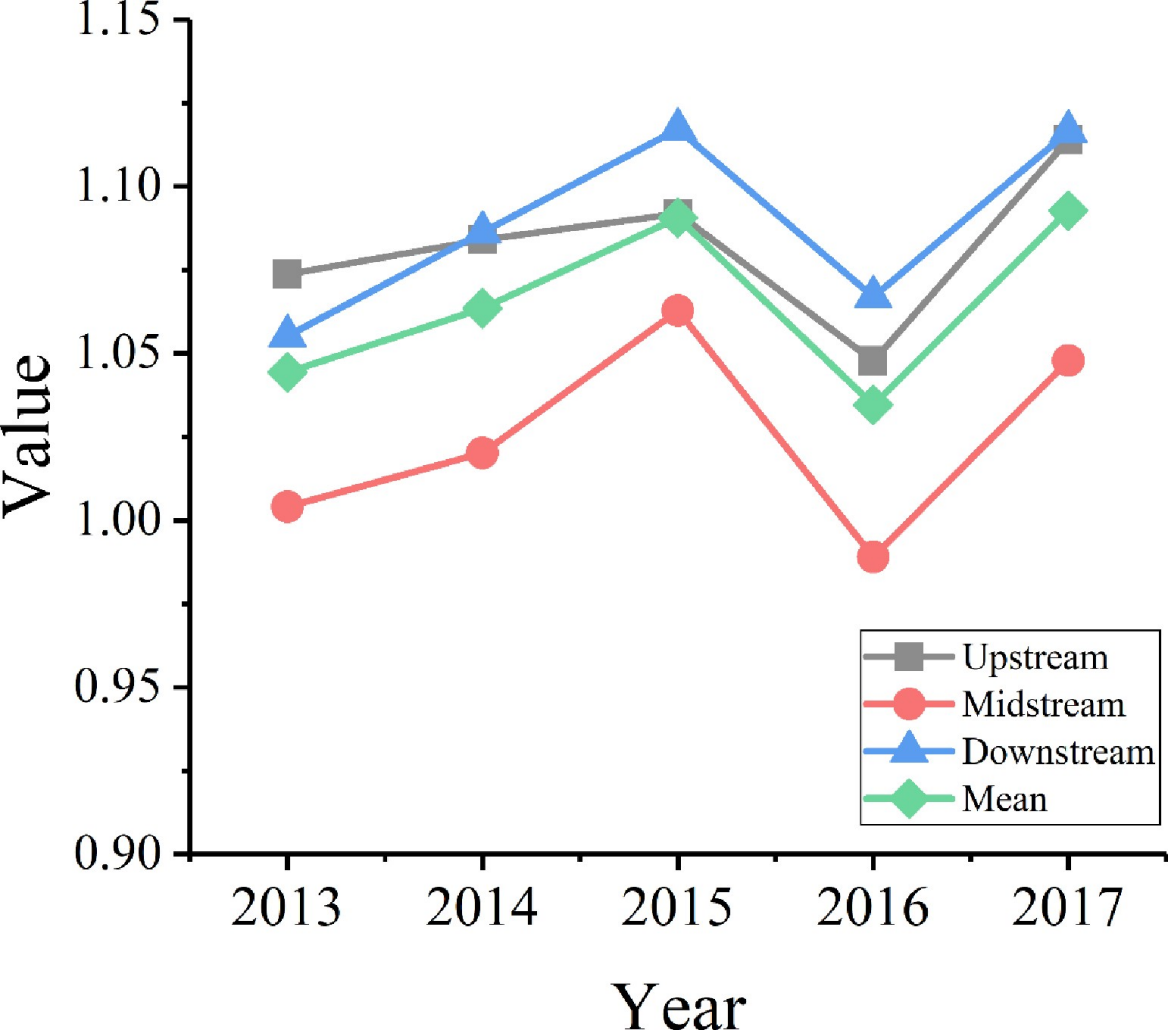
Changing trends in green innovation efficiency in each section of the Yangtze River Economic Belt (2013–2017).

From a spatial perspective (Fig 2), there are significant spatial differences in the green innovation efficiency of the Yangtze River Economic Belt. The downstream regions have the highest average green innovation efficiency. Suzhou, Shanghai, Zhoushan, and Nanjing have high efficiency values, and Suzhou ranks first among all the cities with an average efficiency over the past five years of 1.3731. However, Huzhou, Nantong, Hefei, and Maanshan have values below 1. They have large-scale R&D input, but the output efficiency is low and is accompanied with high levels of pollutant emissions. The upstream cities rank second, and the efficiency values of Lijiang, Kunming, and Chengdu place them among the top ten of all cities. The average efficiency of Liupanshui in the recent five years is only 0.9545, and it ranks last among the upstream cities. Due to limitations in economic development and industrial structure, the midstream cities are always in the last place. Among these cities, the average efficiency of Changsha in the past five years is 1.2418, and it ranks fourth among all the cities. On the other hand, Yichang is placed last with an efficiency of 0.8678.

**Fig 3:**
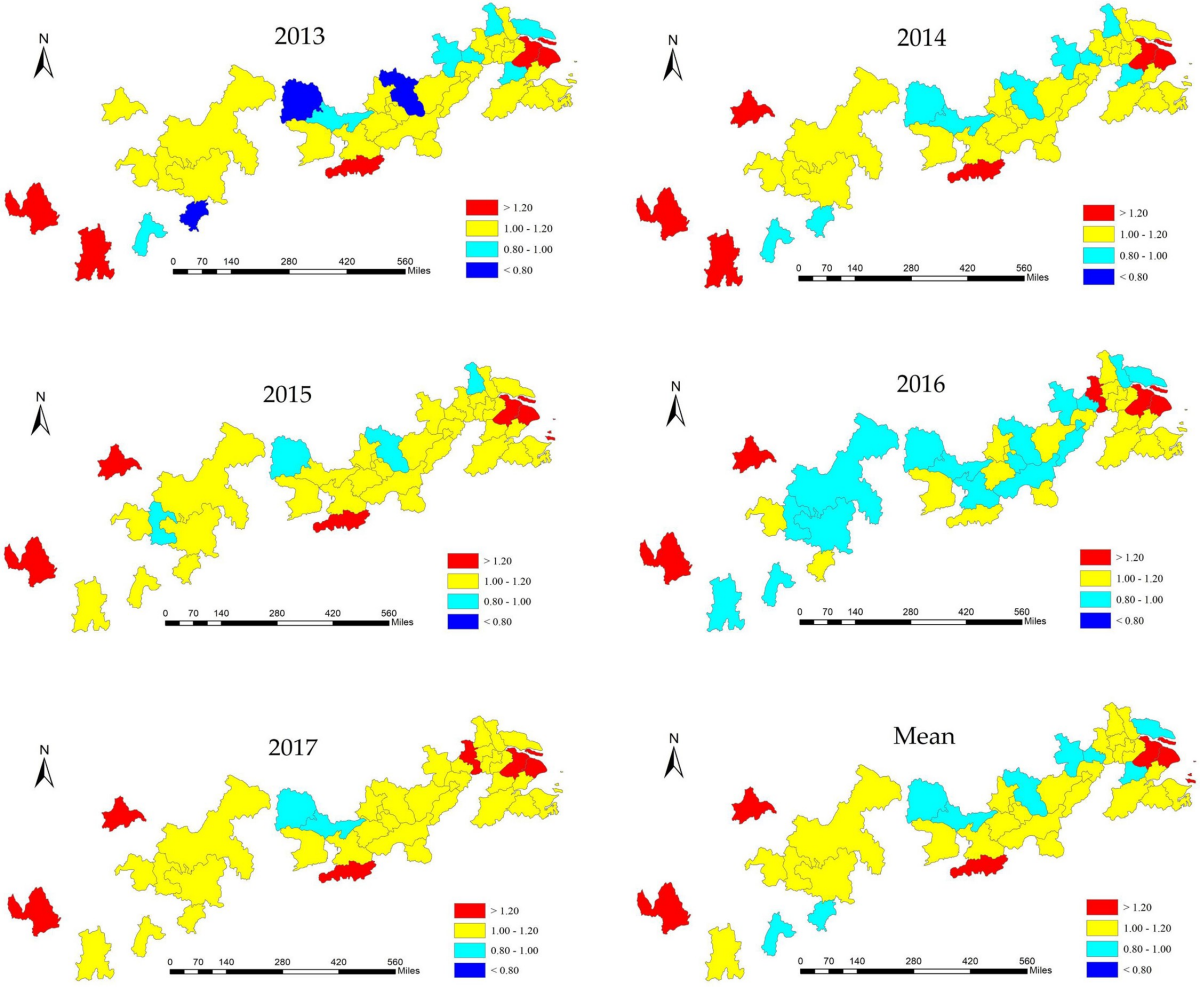
Spatial-temporal differentiation of green innovation efficiency in the Yangtze River Economic Belt (2013–2017).

ArcGIS 10.2 was employed to classify the green innovation efficiency values of cities in the Yangtze River Economic Belt from 2013 to 2017 (Fig 3). As shown in Table 5, since 2013, the green innovation efficiency value of the Yangtze River Economic Belt has remained high, but the spatial distribution is uneven. That is, low-value cities and high-value cities are distributed in various regions of the Yangtze River Economic Belt.

**Table 5:** Green innovation efficiency of different cities in the Yangtze River Economic Belt.

City	2013	2014	2015	2016	2017	Mean	Rank
Lijiang	1.4396	1.2170	1.3298	1.2853	1.2873	1.3118	3
Kunming	1.2836	1.3108	1.0744	0.9709	1.1419	1.1563	8
Liupanshui	0.8297	0.9889	1.0361	0.9025	1.0154	0.9545	39
Guiyang	0.7432	0.9080	1.1193	1.1171	1.0585	0.9892	36
Zunyi	1.0112	1.0091	1.0344	0.9402	1.0518	1.0093	32
Chengdu	1.1185	1.2145	1.2163	1.2390	1.2261	1.2029	6
Yibin	1.0985	1.0289	1.0490	1.0385	1.1070	1.0644	18
Luzhou	1.0801	1.0089	0.9539	0.9730	1.0391	1.0110	31
Chongqing	1.0589	1.0698	1.0134	0.9632	1.0988	1.0408	24
**Upstream**	1.0737	1.0840	1.0919	1.0477	1.1140	1.0823	
Yichang	0.7693	0.8147	0.9384	0.8167	0.9999	0.8678	42
Jingzhou	0.8269	0.8319	1.0499	0.8496	0.9173	0.8951	40
Changde	1.0675	1.0326	1.1108	1.0859	1.0168	1.0627	19
Changsha	1.2854	1.2619	1.2400	1.1509	1.2709	1.2418	4
Yueyang	1.0989	1.0936	1.0941	0.9354	1.0301	1.0504	21
Xianning	1.0796	1.0622	1.0694	1.0110	1.1042	1.0653	17
Wuhan	1.0763	1.1127	1.1689	1.0117	1.0985	1.0936	13
Ezhou	1.0927	1.0890	1.0502	1.0916	1.0816	1.0810	14
Huanggang	0.6476	0.8082	0.9350	0.9829	1.0339	0.8815	41
Huangshi	1.0422	1.0662	1.0052	0.9521	1.0094	1.0150	29
Nanchang	1.0260	1.0105	1.0249	1.0267	1.0189	1.0214	27
Jiujiang	1.0364	1.0592	1.0676	0.9536	0.9932	1.0220	23
**Midstream**	1.0041	1.0202	1.0629	0.9890	1.0479	1.0248	
Anqing	1.1105	1.1022	1.1827	1.1675	1.0992	1.1324	9
Chizhou	1.0282	1.0150	1.0464	0.9280	1.0003	1.0036	33
Tongling	1.0190	1.0610	1.0997	0.9555	1.0234	1.0317	26
Hefei	0.8329	0.9950	1.0419	0.8157	1.1009	0.9573	38
Wuhu	1.0403	1.0802	1.1001	1.0689	1.0825	1.0744	15
Maanshan	0.8975	0.9976	1.0756	0.9501	1.0623	0.9966	34
Nanjing	1.0990	1.1418	1.1828	1.2261	1.2126	1.1725	7
Zhenjiang	1.0556	1.1186	1.1305	1.1271	1.1148	1.1093	11
Yangzhou	0.9141	0.9503	0.9964	1.0085	1.1999	1.0139	30
Taizhou	1.0321	1.0349	1.0333	0.9882	1.1089	1.0395	25
Changzhou	1.0044	1.0122	1.0117	1.0091	1.0494	1.0174	28
Wuxi	1.0836	1.0276	1.0411	1.0361	1.1522	1.0681	16
Nantong	0.9998	1.0090	1.0208	0.8513	1.0607	0.9883	37
Hangzhou	1.1136	1.1748	1.1341	1.0414	1.0393	1.1006	12
Shaoxing	1.0177	1.0473	1.1214	1.0902	1.0260	1.0605	20
Huzhou	0.8070	0.8813	1.0957	1.0883	1.0984	0.9941	35
Jiaxing	1.1207	1.1996	1.1184	1.0321	1.0987	1.1139	10
Suzhou	1.3617	1.2749	1.3537	1.4888	1.3867	1.3731	1
Shanghai	1.4127	1.5085	1.2187	1.3365	1.3199	1.3592	2
Ningbo	1.0504	1.0581	1.0575	1.0389	1.0175	1.0445	22
Zhoushan	1.1592	1.1224	1.3973	1.1577	1.1910	1.2055	5
**Downstream**	1.0552	1.0863	1.1171	1.0669	1.1164	1.0884	
Mean	1.0446	1.0669	1.0962	1.0406	1.0987	1.0694	

### Dynamic green innovation efficiency

**Fig 4:**
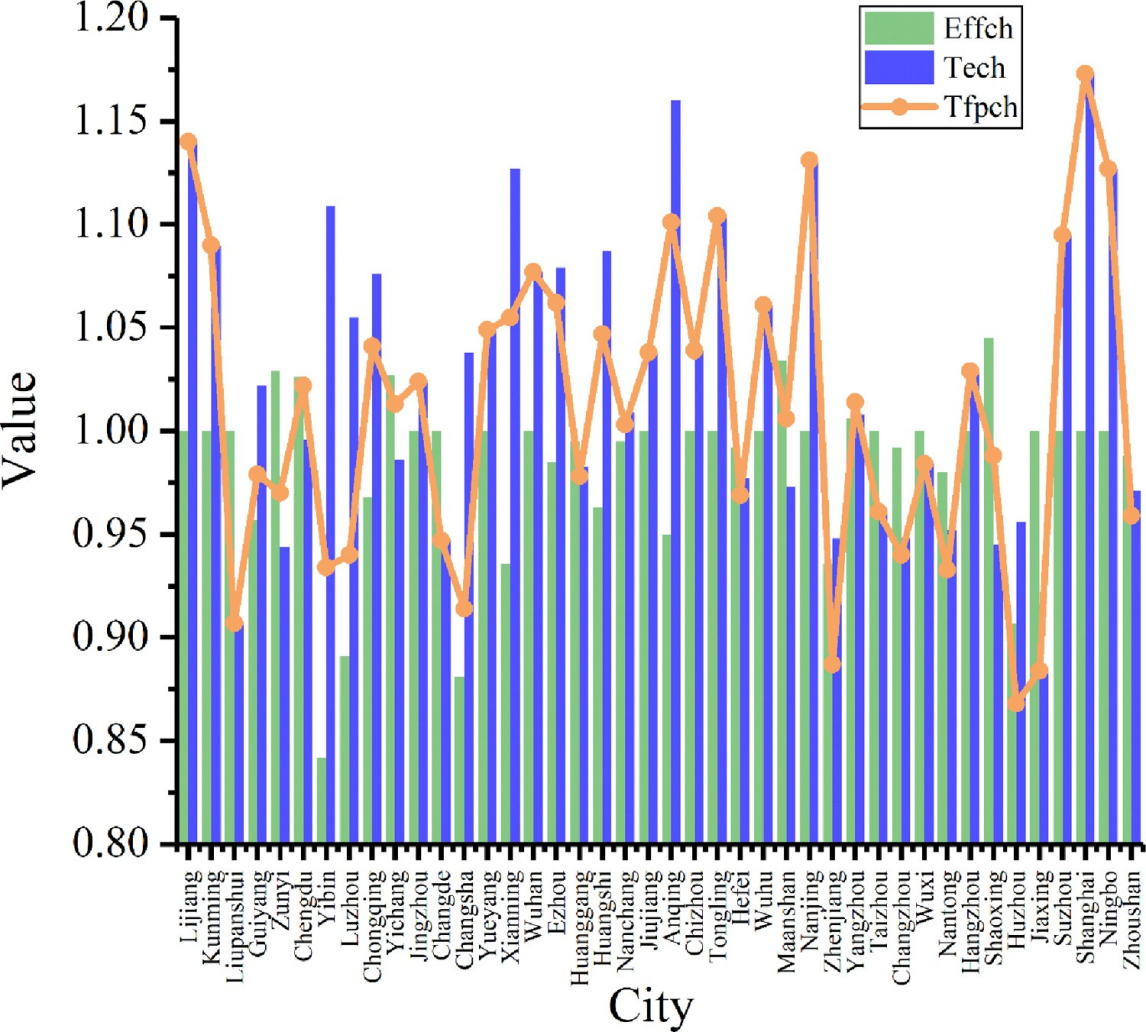
ML index and decomposition of green innovation efficiency in the Yangtze River Economic Belt (2013–2017).

**Fig 5:**
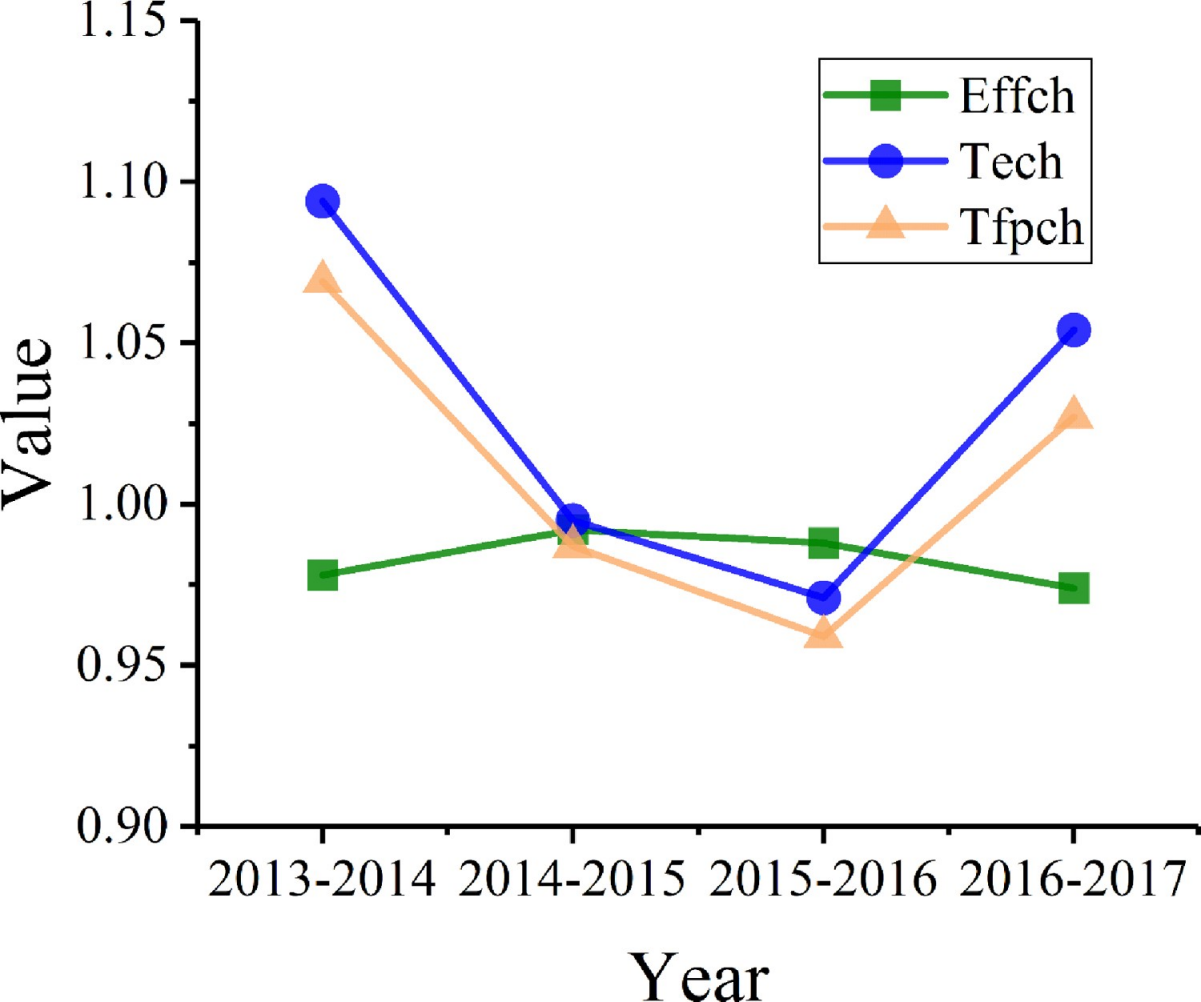
Decomposition of green innovation efficiency in the Yangtze River Economic Belt based on a time-varying trend.

In order to further analyze the changes in green innovation efficiency and to identify the reasons for the change, the ML index of green innovation efficiency was calculated with the panel data of 42 cities along the Yangtze River from 2013 to 2017. The data include the total factor productivity index (Tfpch) by city (Fig 4) and by year (Table 6 and Fig 5).

**Table 6:** Annual ML index and decomposition of green innovation efficiency in the Yangtze River Economic Belt.

Year	Effch	Tech	Tfpch
2013–2014	0.978	1.094	1.069
2014–2015	0.992	0.995	0.987
2015–2016	0.988	0.971	0.959
2016–2017	0.974	1.054	1.027
Mean	0.983	1.029	1.011

As shown in Fig 4, the average total factor productivity of green innovation in the 42 major cities in the Yangtze River Economic Belt from 2013 to 2017 was 1.011; with an average annual growth rate of 1.1%, the overall green innovation total factor productivity was on the rise. The total factor productivity of green innovation was lower than 1 in 42.86% of the cities, among which Huzhou had the lowest total factor productivity (0.868), with an average annual decline of 13.2%. Suzhou and Nanchang also showed a decline in total factor productivity. Among all the cities, Shanghai ranked first with an annual growth rate of 14.3%, and Nanjing and Wuhan had an annual growth rate of over 5%.

According to Eq (3), the ML index is decomposed into technology efficiency change (Effch) and technical change (Tech). From the results of the decomposition, the average technology efficiency change of the 42 cities in 2013–2017 was 0.983. Shaoxing ranked first with a change of 1.045, and only Chengdu, Yangzhou, Yichang, Zunyi, and Maanshan had change values lower than 1. The average technical change was 1.029. Shanghai ranked first with an average annual growth rate of 17.3%, and Nanjing and Ningbo were among the top five. In general, the improvement of technical change has promoted increase in factor productivity.

As illustrated in Table 6, the average values of total factor productivity and technical change are more than 1. This indicates that overall efficiency and technical change showed an increase from 2013 to 2017. In particular, it appears that technical change, with an average annual growth rate of 2.9%, has become the main driving force for the continuous growth of total factor productivity. The average technology efficiency change is less than 1, which indicates that technology efficiency change showed a downward trend over the five years. As shown in Fig 4, technology efficiency change and technical change show a trend that is indicative of reverse development.

### Spatial correlation analysis

#### Global spatial correlation analysis

This paper uses GEODA to examine the spatial correlation of green innovation efficiency in 42 cities of the Yangtze River Economic Belt from 2013 to 2017. The results are shown in Table 7. According to this table, the overall Moran’s I index of green innovation efficiency of the Yangtze River Economic Belt from 2013 to 2017 is positive and passes the significance test at the 10% level. This indicates that the green innovation efficiency of neighboring cities has a spatial agglomeration effect.

**Table 7:** Global autocorrelation analysis of green innovation efficiency in the Yangtze River Economic Belt (2013–2017).

Year	Moran’s I	P-value
2013	0.620	0.003
2014	0.712	0.001
2015	0.204	0.020
2016	0.134	0.060
2017	0.378	0.042

#### Local spatial correlation analysis

**Fig 6:**
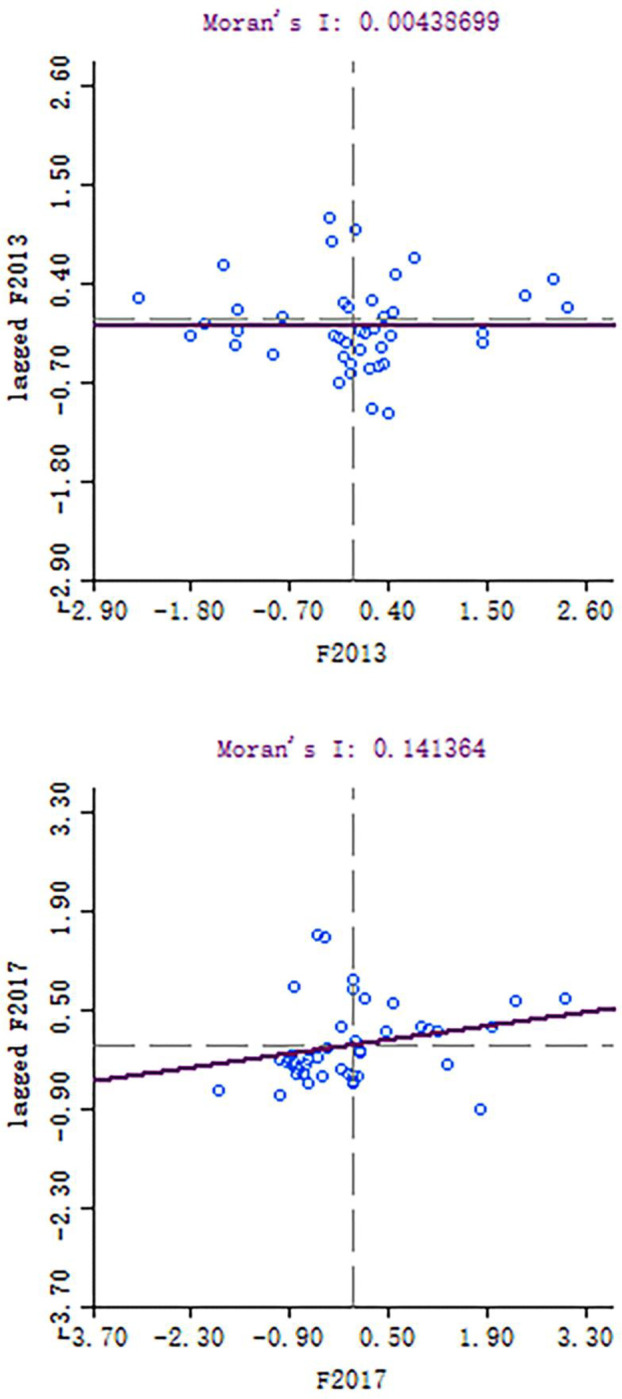
Moran scatter plot of green innovation efficiency in the Yangtze River Economic Belt (2013, 2017).

Global spatial correlation analysis was employed to examine the overall spatial agglomeration effect of green innovation efficiency in the 42 cities of the Yangtze River Economic Belt. In order to further discriminant cities surrounding the existence of spatial agglomeration phenomenon, this paper uses GEODA to calculate the green innovation efficiency of local Moran scatter diagram in Yangtze River Economic Belt from 2013 to 2017 (Fig 6), and the Moran scatter diagram of each quadrant changes the condition of a city are analyzed (Table 8).

**Table 8:** Results of the Moran scatter chart (2013, 2017).

City	2013	2017	City	2013	2017	City	2013	2017
Lijiang	HH	HH	Xianning	HH	HL	Zhenjiang	HL	HH
Kunming	HL	HH	Wuhan	HL	LL	Yangzhou	LH	HH
Liupanshui	LH	LL	Ezhou	HL	LL	Taizhou	LL	HL
Guiyang	LL	LL	Huanggang	LH	LL	Changzhou	LH	LH
Zunyi	LL	LL	Huangshi	LL	LL	Wuxi	HL	HH
Chengdu	HH	HL	Nanchang	LH	LL	Nantong	LH	LH
Yibin	HL	HL	Jiujiang	LH	LL	Hangzhou	HL	LL
Luzhou	HL	LL	Anqing	HL	HL	Shaoxing	LL	LL
Chongqing	HL	HL	Chizhou	LL	LL	Huzhou	LH	LH
Yichang	LL	LL	Tongling	LL	LL	Jiaxing	HH	HH
Jingzhou	LL	LL	Hefei	LL	HH	Suzhou	HH	HH
Changde	HL	LL	Wuhu	LL	LH	Shanghai	HH	HH
Changsha	HL	HL	Maanshan	LL	LL	Ningbo	HH	LH
Yueyang	HH	LL	Nanjing	HL	HH	Zhoushan	HH	HH

As shown in Table 8, in 2013, a total of 9 cities exhibited “HH” agglomeration, including Lijiang, Chengdu, Suzhou, and Shanghai. Further, 13 cities exhibited “HL” agglomeration, including Chongqing, Wuhan, Changsha, Nanjing, and Hangzhou, which accounted for 30.95% of the total cities. Further, the number of “LH” agglomeration and “LL” agglomeration cities was 8 and 11, respectively. In 2017, the number of “HH” agglomeration cities increased to 11, and they accounted for 26.2% of the total number of cities. The number of “HL” agglomeration and “LH” agglomeration cities was 7 and 5, respectively, and the number of “LL” agglomeration cities increased to 19 and included Wuhan and Hangzhou. From a spatial perspective, “LL” agglomeration cities are mainly in the middle reaches of the Yangtze River, for example, Huangshi, Jingzhou, and Yichang, and “HH” agglomeration cities are mainly in the lower reaches of the Yangtze River, for example, Shanghai, Suzhou, and Ningbo.

### Coupling coordination degree analysis

**Fig 7:**
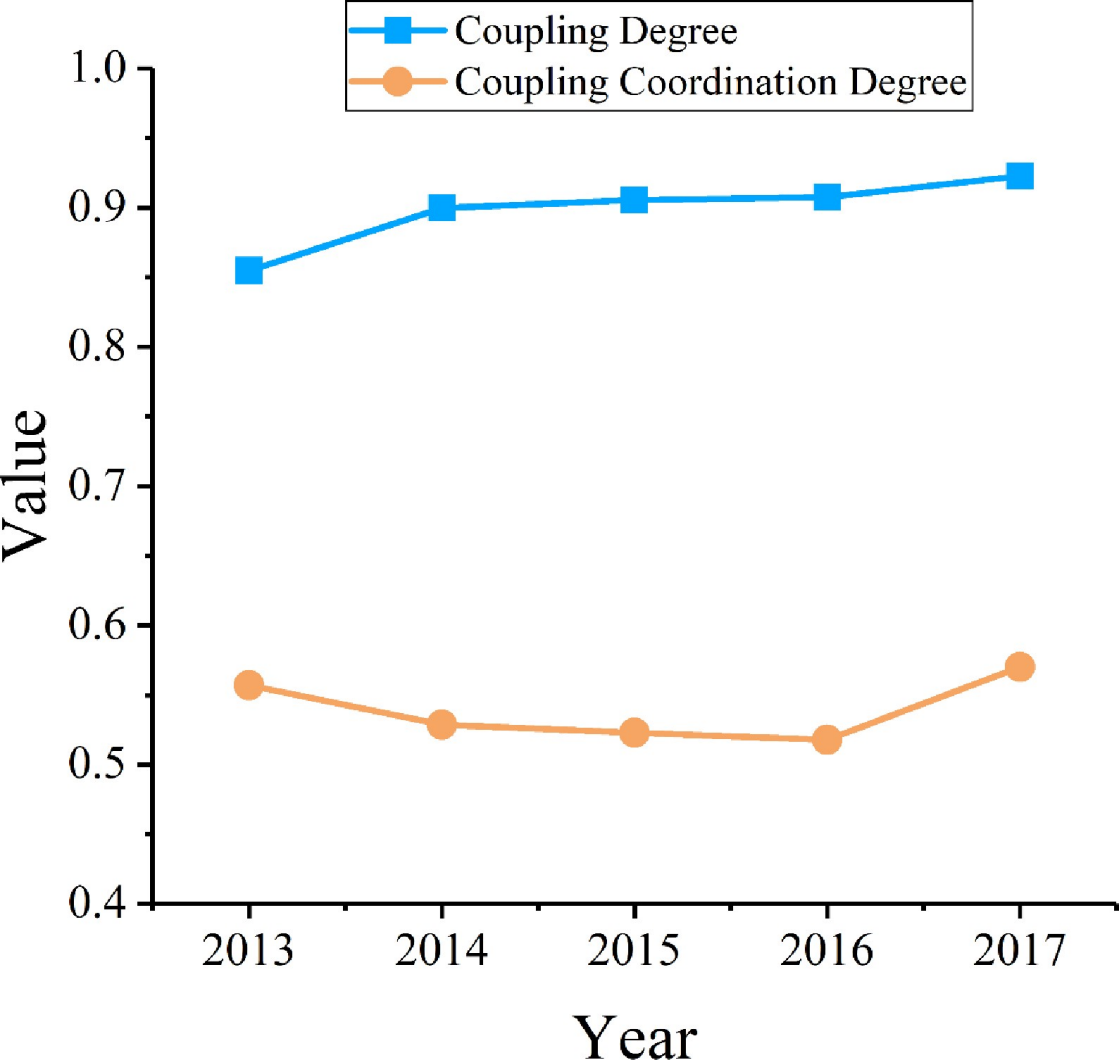
Trends in coupling coordination degree.

After exploring green innovation efficiency and its spatial effect in the Yangtze River Economic Belt, the next step was to analyze the synergetic effect of green innovation efficiency and economic development with the coupling coordination degree model. Accordingly, the coupling coordination degree between green innovation efficiency and the development of high-tech industries in the Yangtze River Economic Belt from 2013 to 2017 was calculated (Fig 7). The mean value of coupling degree C fluctuated between 0.85 and 0.95, and the mean value of coupling coordination degree D was generally between 0.5 and 0.6 (Table 9). This means that the area is in the basic coordination stage, and there is still scope for future development.

**Table 9:** Coupling coordination degree of green innovation efficiency and high-tech industries development in the Yangtze River Economic Belt (2013–2017).

Coupling Coordination Degree	2013	2014	2015	2016	2017
Lijiang	0.5623	0.5001	0.5429	0.5194	0.5334
Kunming	0.5740	0.5874	0.4987	0.4881	0.6091
Liupanshui	0.4206	0.4283	0.4154	0.3862	0.4202
Guiyang	0.3822	0.3929	0.4670	0.4912	0.4701
Zunyi	0.4805	0.4418	0.4212	0.4147	0.5032
Chengdu	0.6773	0.6664	0.6537	0.7401	0.7710
Yibin	0.5146	0.4629	0.4435	0.4783	0.5217
Luzhou	0.5157	0.4524	0.3598	0.4368	0.5038
Chongqing	0.7026	0.7344	0.6641	0.4696	0.6860
Yichang	0.4307	0.3732	0.3807	0.3785	0.4764
Jingzhou	0.4307	0.3542	0.4478	0.3666	0.3434
Changde	0.5123	0.4727	0.4941	0.5032	0.4849
Changsha	0.7030	0.7539	0.7770	0.7366	0.9313
Yueyang	0.5528	0.5361	0.5268	0.4730	0.5346
Xianning	0.4994	0.4651	0.4494	0.4509	0.4938
Wuhan	0.6597	0.6792	0.7152	0.6417	0.7634
Ezhou	0.5092	0.4807	0.4419	0.4864	0.4864
Huanggang	0.3252	0.3301	0.3312	0.4470	0.4674
Huangshi	0.5090	0.4878	0.4143	0.4322	0.4491
Nanchang	0.5439	0.5103	0.4601	0.5057	0.5287
Jiujiang	0.5087	0.4929	0.4742	0.4508	0.5363
Anqing	0.5256	0.5002	0.5191	0.5117	0.5048
Chizhou	0.4822	0.4456	0.4317	0.4083	0.4209
Tongling	0.5289	0.5431	0.4803	0.4379	0.4504
Hefei	0.5421	0.5966	0.5370	0.4151	0.6810
Wuhu	0.5819	0.5873	0.5409	0.5604	0.6108
Maanshan	0.4823	0.4760	0.4449	0.4270	0.4688
Nanjing	0.5813	0.5356	0.8736	0.8975	0.7133
Zhenjiang	0.5774	0.5282	0.5362	0.5065	0.8318
Yangzhou	0.5120	0.4440	0.4427	0.4799	0.6064
Taizhou	0.6042	0.4639	0.4971	0.5090	0.4844
Changzhou	0.5771	0.4868	0.4221	0.4624	0.5217
Wuxi	0.5789	0.4992	0.4339	0.4645	0.5271
Nantong	0.6057	0.4792	0.4992	0.4239	0.5361
Hangzhou	0.6320	0.6270	0.6188	0.5935	0.6384
Shaoxing	0.6016	0.4560	0.5299	0.5329	0.5217
Huzhou	0.4293	0.4058	0.5009	0.5158	0.5314
Jiaxing	0.6681	0.6850	0.5513	0.5336	0.6060
Suzhou	0.9771	0.9146	0.7745	0.7936	0.8229
Shanghai	0.7455	0.8350	0.7773	0.8751	0.7854
Ningbo	0.5974	0.5792	0.5527	0.5755	0.6516
Zhoushan	0.5422	0.5104	0.6110	0.5225	0.5123

**Fig 8:**
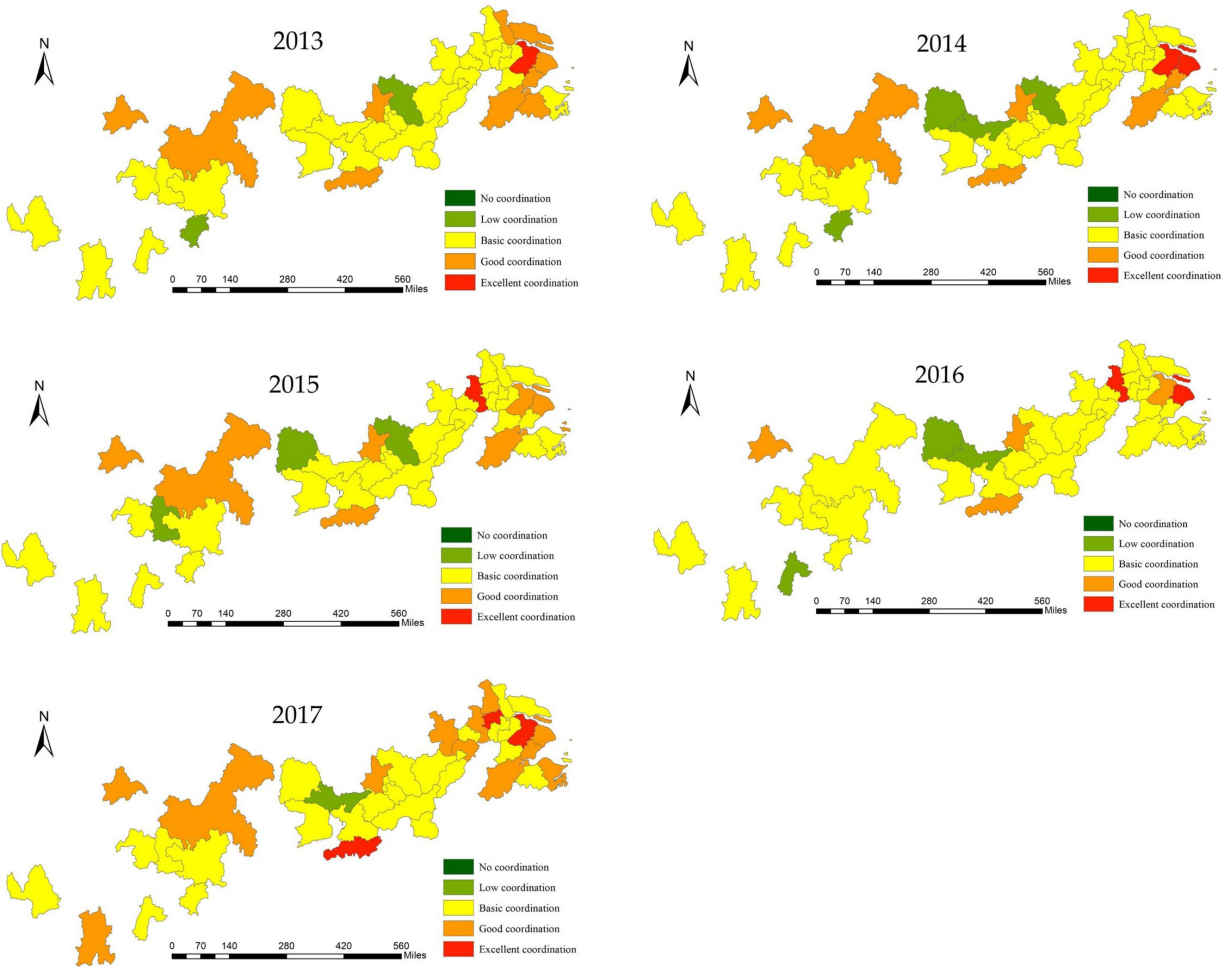
Spatial pattern evolution of the coupling coordination degree of green innovation efficiency and high-tech industries development (2013–2017).

According to the standard classification of coupling coordination degree (Table 4), Arcgis10.2 was used to conduct spatial classification of the coupling coordination degree between green innovation efficiency and high-tech industry development (Fig 8). As seen from the figure, from 2013 to 2017, there were no uncoordinated cities along the Yangtze River. In 2013, the average coupling coordination degree of cities in the Yangtze River Economic Belt was 0.556. The coupling coordination degree for Suzhou was 0.977, which means that it was the only city that achieved excellent coordination. Ten other cities showed good coordination, including Chengdu, Chongqing, Changsha, Wuhan, Hangzhou, and Shanghai, and only Huanggang and Guiyang showed low coordination. By 2017, the average coupling coordination degree of all the cities increased to 0.570, and Changsha, Zhenjiang, and Suzhou showed excellent coordination. Twelve other cities, including Kunming, Chengdu, Chongqing, Wuhan, Hefei, Nanjing, Hangzhou, Shanghai, and Ningbo, achieved good coordination. The coupling coordination degree of Jingzhou was 0.343, and this makes it the only city with low coordination.

## Discussion

In recent years, China has strongly supported the promotion of high-quality development in the Yangtze River Economic Belt. Therefore, it is important for the government to formulate development policies to comprehensively explore the current situation with regard to green innovation and efficiency development in various cities.

### Changes in green innovation efficiency and its driving factors

China focused on improving the development of the Yangtze River Economic Belt to meet the national strategic level and announced a series of important policies to promote green innovation in the region from 2012, such as opinions on the full implementation of the river system [60], the plan for industrial transformation and upgrade driven by innovation in the Yangtze River Economic Belt [61], and guiding opinions on improving the relevant policies of the sewage treatment charging mechanism of the Yangtze River Economic Belt [62]. In keeping with these policy changes, the static and dynamic green innovation efficiency results in this study show a trend towards growth. As a result of the Yangtze River Economic Development Symposium, major cities along the Yangtze River adjusted their industrial structure on a large scale and reduced high-polluting enterprises, and the green innovation efficiency declined from 2015 to 2016, as reported by Liu [63] and Yi [23]. However, the measured green innovation efficiency in Liu’s study did not consider differences in the input-output indicators or undesirable outputs. According to the present results, with regard to the development of different cities, Suzhou and Shanghai show a high level of economic strength and scientific research strength, and have achieved the transformation and upgrade of high-pollution industries at an earlier stage. Green innovation efficiency has always been in the top two. Although Lijiang, Kunming, Yibin, and other western cities show weak innovation development, they value environmental protection and rank high in green innovation efficiency. Additionally, Chongqing, Chengdu, Nanjing, and Wuhan have achieved effective levels of green innovation efficiency earlier due to their economic strength and scientific research advantages. In Nantong and Huzhou, environmental protection was not considered in the early pursuit of economic development, but this resulted in a low level of green innovation efficiency in 2013. However, in light of the new round of domestic industrial transfer, these cities quickly adjusted their industrial structure and improved their green innovation efficiency. Among the cities at the bottom, in Yichang and Jingzhou, the chemical industries account for a large proportion of the economic development and are located at the edge of the Chengdu-Chongqing city cluster and Wuhan metropolitan area. Poor talent attraction, infrastructure, and green innovation efficiency are some of the reasons why these cities are at the bottom of the list.

### Spatial correlation analysis of green innovation efficiency

According to the results of spatial correlation analysis, green innovation efficiency in the Yangtze River Economic Belt showed significant and positive spatial correlation, but the agglomeration patterns are different in different sections of the Yangtze River. In agreement with these findings, Xu [25] also found that green innovation efficiency has a spatially positive correlation in the Yangtze River Economic Belt. The Yangtze River Economic Belt covers a wide area across which there is a significant gap in regional development. The upstream cities attach importance to environmental protection, while the downstream cities attach importance to economic development. Further, the development characteristics of the midstream cities are not obvious. In the local spatial autocorrelation analysis, cities in the middle reaches of the Yangtze River, such as Yueyang and Changde, are dominated by resource-intensive industries such as chemical industries. They consume large amounts of energy resources and cause serious environmental pollution and are characterized by “LL” agglomeration. The cities on the upper reaches of the Yangtze River, such as Kunming, Chengdu, and Chongqing, as the capital cities of the western region, have absorbed the resources of the surrounding cities on a large scale, and their own economic development has been rapid. However, this has seriously affected the development of other surrounding cities and, thereby, resulted in “HL” agglomeration. According to Lijiang, the development of traditional manufacturing is difficult because of inconvenience in transportation. Therefore, the government has vigorously promoted the development of technology-intensive industries to promote economic development, and this explains the “HH” agglomeration. Cities in the lower reaches of the Yangtze river, such as Shanghai, Suzhou, and Ningbo, are economically developed cities. These cities are more attractive to scientific research talents, and have realized industrial transformation and upgrading at an earlier stage, achieved high-quality economic development, and formed an “HH” agglomeration. Hangzhou and Shaoxing are both cities in the lower reaches of the Yangtze River, but their talents are far less attractive than those of Shanghai. Hangzhou has vigorously developed the e-commerce industry and Shaoxing has a large-scale chemical industry. The high-tech industries development atmosphere is poor, resulting in their “LL” agglomeration.

### Coupling coordination relationship between green innovation efficiency and high-tech industries development

According to the results of coupling coordination degree analysis, the coupling coordination degree between green innovation efficiency and high-tech industries development is basic, and the interaction between green innovation and economic development is not obvious. Improving green innovation efficiency can promote economic development, especially through innovation in high-tech industries. Observing development from the perspective of coupling coordination would be beneficial in terms of formulating different policies based on the current situation. The upstream cities Lijiang, Liupanshui, and Kunming are located in the west of China. Their geographical location is poor and scientific research foundation is weak, and this has led to the lag in the development of high-tech industries. The populations of midstream and downstream cities, such as Wuhan, Shanghai, and Nanjing, are concentrated, and there is a need for long-term industrial development that is beneficial to the ecological environment. However, these cities lag behind in terms of green innovation efficiency, in accordance with the results of Liu [64].

### Limitations

Although this paper studies green development efficiency, its driving factors, spatial distribution, and the coupling coordination relationship between the Yangtze River Economic Belt and high-tech industries with a variety of methods, it still has the following limitations. First, there is no empirical research on the influencing factors associated with green innovation efficiency in the Yangtze River Economic Belt. Therefore, it is impossible to propose effective improvement strategies in a targeted manner. Second, the input-output indicators need to be improved, because economic development will bring new innovations to green innovation that will require the selection of new indicators in the future.

## Conclusions

Green innovation is conducive to the high-quality development of China’s economy. Therefore, this paper studies 42 major cities in the Yangtze River Economic Belt as the research object. The static and dynamic efficiency of green innovation is measured, and a comprehensive analysis of green innovation efficiency from the two dimensions of spatial effects and synergistic effect is presented. The research conclusions are as follows:

From 2013 to 2017, the average values of green innovation efficiency in the Yangtze River Economic Belt increased from 1.0446 to 1.0987. Due to differences in geographical location and resource endowment of each city, the development speed of green innovation in cities along the Yangtze River was different. The average value of green innovation efficiency in the downstream cities was the highest at 1.0884; they were followed by the upstream cities with an average value of 1.0823 and the midstream cities with the lowest value of 1.0248.

The total factor productivity of green innovation in the Yangtze River Economic Belt increased by 1.1% per year from 2013 to 2017. Technical change, with an average annual growth rate of 2.9%, was the main driving force of green innovation total factor productivity, but technology efficiency change fell by an average of 1.7% a year.

All cities in the Yangtze River Economic Belt present a significant positive spatial correlation: “HH” agglomeration cities are mainly distributed in the lower reaches of the Yangtze River; “LL” agglomeration cities are mainly distributed in the middle reaches of the Yangtze River; and the capital cities in the upper reaches of the Yangtze River exhibit “HL” agglomeration.

The coupling coordination degree between green innovation efficiency and the development of high-tech industries in cities of the Yangtze River Economic Belt is between 0.5 and 0.6, which indicates that this region in the basic coordination stage. Additionally, the coupling coordination degree presents a fluctuating rising trend. Overall, the synergies between green innovation efficiency and economic development need to be improved.

Based on the research results, in order to effectively improve the green innovation efficiency of the Yangtze River Economic Belt and promote the healthy development of economy and environment, the following suggestions are proposed:

While promoting overall green innovative development in the Yangtze River Economic Belt, more attention should be paid to coordinated development in different sections of the Yangtze River. The upstream cities can strengthen the construction of infrastructure, and improve the economic level and innovation capacity of all the cities. While promoting the transformation of green and innovative development, midstream cities can consistently coordinate the relationship between economic growth and ecological governance, strengthen the transformation of innovative achievements and the introduction of new and advanced technologies, and realize sustainable development. Downstream cities can invest large amounts of capital and cooperate with each other to promote ecological and environmental governance, so as to achieve harmonious coexistence of economy and society.

The green innovation technical changes of the Yangtze River Economic Belt has greatly improved, but technology efficiency change is still a shortcoming of development. Therefore, technology efficiency change should be actively guided by cities with abundant innovation resources to improve the utilization rate of innovation factors, improve the ability to transform innovation achievements, and reduce the consumption of innovation resources.

The spatial spillover effect of high-value cities with green innovation efficiency should be used to broaden the channels for the diffusion of innovation achievements, and policies and markets should be designed to promote the coordinated development of green innovation in the Yangtze River Economic Belt.

Cities with good economic development should use their capital advantages to increase R&D and introduce advanced innovations to promote the development of green innovation; at the same time, cities with better green innovation development can strengthen their ability to transform innovations, promote urban economic development and, ultimately, promote green innovation alongside economic development.

## Appendix

**Table A1:** Coupling degree of green innovation efficiency and high-tech industries development in the Yangtze River Economic Belt (2013–2017).

Coupling Degree	2013	2014	2015	2016	2017
Lijiang	0.5750	0.6895	0.6085	0.6517	0.6257
Kunming	0.6903	0.7620	0.9246	0.9684	0.9392
Liupanshui	0.8654	0.8463	0.8691	0.9352	0.8910
Guiyang	0.9398	0.9280	0.7756	0.7800	0.8795
Zunyi	0.7483	0.8403	0.8853	0.9113	0.9430
Chengdu	0.9494	0.9457	0.9194	0.9812	0.9886
Yibin	0.7286	0.8514	0.8899	0.8642	0.8731
Luzhou	0.7480	0.8618	0.9985	0.8925	0.9638
Chongqing	0.9904	0.9779	0.8625	0.9571	0.9988
Yichang	0.9695	0.9695	0.9543	0.9429	0.9917
Jingzhou	0.8896	0.9992	0.8956	0.9969	0.9865
Changde	0.7537	0.8646	0.8463	0.8437	0.9779
Changsha	0.8818	0.9833	0.9904	0.9999	0.9941
Yueyang	0.7988	0.8929	0.9286	0.9888	0.9949
Xianning	0.7165	0.8069	0.8514	0.8550	0.8292
Wuhan	0.9567	0.9980	0.9967	0.9918	0.9661
Ezhou	0.7235	0.8012	0.8840	0.8044	0.8605
Huanggang	0.9984	0.9963	0.9958	0.8942	0.9275
Huangshi	0.7725	0.8457	0.9507	0.9203	0.9522
Nanchang	0.8511	0.9498	0.9667	0.9245	0.9986
Jiujiang	0.7778	0.8642	0.9007	0.9462	0.9182
Anqing	0.7388	0.8218	0.7623	0.7583	0.8588
Chizhou	0.7334	0.8384	0.8741	0.9230	0.9318
Tongling	0.8327	0.9369	0.8433	0.9239	0.9240
Hefei	0.9985	0.9990	0.9978	0.8683	0.9997
Wuhu	0.8957	0.9659	0.9369	0.9468	0.9939
Maanshan	0.8863	0.9194	0.8273	0.9149	0.8689
Nanjing	0.8456	0.8384	0.9646	0.9770	0.9647
Zhenjiang	0.8764	0.8520	0.8812	0.7971	0.9358
Yangzhou	0.9143	0.9385	0.9936	0.9103	0.8627
Taizhou	0.9300	0.8445	0.9865	0.9710	0.8018
Changzhou	0.9190	0.9162	0.9461	0.8798	0.9669
Wuxi	0.8549	0.9153	0.8920	0.8418	0.8047
Nantong	0.9538	0.9089	0.9976	0.9808	0.9657
Hangzhou	0.9047	0.9340	0.9715	0.9914	0.9805
Shaoxing	0.9375	0.8099	0.8868	0.8879	0.9922
Huzhou	0.9169	0.9867	0.8878	0.8626	0.9030
Jiaxing	0.9396	0.9694	0.9221	0.9530	0.9806
Suzhou	0.9989	0.9843	0.9170	0.9019	0.9286
Shanghai	0.8630	0.9383	0.9971	0.9992	0.9430
Ningbo	0.9080	0.9746	0.9947	0.9832	0.9339
Zhoushan	0.7276	0.8160	0.6553	0.7902	0.7140

**Table A2:** Coordinate values of green innovation efficiency and high-tech industries development in the Yangtze River Economic Belt (2013–2017).

T	2013	2014	2015	2016	2017
Lijiang	0.5500	0.3627	0.4843	0.4140	0.4547
Kunming	0.4773	0.4527	0.2690	0.2461	0.3950
Liupanshui	0.2045	0.2168	0.1985	0.1595	0.1982
Guiyang	0.1554	0.1663	0.2813	0.3093	0.2512
Zunyi	0.3085	0.2323	0.2004	0.1887	0.2685
Chengdu	0.4832	0.4696	0.4648	0.5582	0.6013
Yibin	0.3634	0.2516	0.2210	0.2647	0.3118
Luzhou	0.3555	0.2375	0.1297	0.2138	0.2633
Chongqing	0.4984	0.5515	0.5114	0.2304	0.4711
Yichang	0.1914	0.1436	0.1519	0.1520	0.2288
Jingzhou	0.2085	0.1256	0.2239	0.1348	0.1196
Changde	0.3482	0.2585	0.2885	0.3001	0.2405
Changsha	0.5604	0.5780	0.6095	0.5426	0.8724
Yueyang	0.3826	0.3219	0.2989	0.2263	0.2873
Xianning	0.3481	0.2681	0.2372	0.2378	0.2940
Wuhan	0.4548	0.4623	0.5133	0.4152	0.6032
Ezhou	0.3584	0.2884	0.2209	0.2941	0.2750
Huanggang	0.1059	0.1094	0.1101	0.2234	0.2355
Huangshi	0.3354	0.2814	0.1805	0.2030	0.2119
Nanchang	0.3475	0.2742	0.2190	0.2767	0.2799
Jiujiang	0.3327	0.2812	0.2496	0.2148	0.3132
Anqing	0.3740	0.3044	0.3535	0.3453	0.2967
Chizhou	0.3170	0.2368	0.2132	0.1806	0.1902
Tongling	0.3359	0.3148	0.2735	0.2075	0.2195
Hefei	0.2943	0.3563	0.2890	0.1984	0.4639
Wuhu	0.3781	0.3571	0.3122	0.3317	0.3753
Maanshan	0.2624	0.2465	0.2392	0.1993	0.2529
Nanjing	0.3996	0.3422	0.7912	0.8244	0.5274
Zhenjiang	0.3804	0.3275	0.3262	0.3219	0.7393
Yangzhou	0.2867	0.2101	0.1972	0.2530	0.4263
Taizhou	0.3926	0.2549	0.2505	0.2668	0.2926
Changzhou	0.3625	0.2586	0.1883	0.2430	0.2815
Wuxi	0.3920	0.2723	0.2110	0.2563	0.3453
Nantong	0.3846	0.2527	0.2498	0.1832	0.2976
Hangzhou	0.4415	0.4208	0.3942	0.3553	0.4157
Shaoxing	0.3861	0.2567	0.3166	0.3199	0.2743
Huzhou	0.2010	0.1669	0.2827	0.3084	0.3128
Jiaxing	0.4750	0.4841	0.3296	0.2988	0.3745
Suzhou	0.9557	0.8499	0.6541	0.6983	0.7293
Shanghai	0.6440	0.7430	0.6060	0.7664	0.6542
Ningbo	0.3930	0.3441	0.3071	0.3369	0.4547
Zhoushan	0.4041	0.3193	0.5697	0.3455	0.3675

## Supporting information

S1 Raw data (DOCX)
