# Supplementary material for: Spatial analysis, coupling coordination, and efficiency evaluation of green innovation: A case study of the Yangtze River Economic Belt
Source: PLoS One. 2020 Dec 9;15(12):e0243459. doi: 10.1371/journal.pone.0243459 (PMC7725298; doi:10.1371/journal.pone.0243459)
Supplement: S1 Raw data — (DOCX) [file pone.0243459.s001.docx]

| City | Year | R&D personnel full-time equivalent | Internal R&D expenditures | Fiscal expenditure on science and technology | Unit GDP energy consumption | Number of unauthorized patents | "Green governance" inflation pressure |  |
| --- | --- | --- | --- | --- | --- | --- | --- | --- |
| Lijiang | 2013 | 1023 | 18325 | 18924 | 1.566286352 | 127 | 0.027 |  |
| Kunming | 2013 | 5757 | 199235.2 | 121709 | 0.591574414 | 2985 | 0.039 |  |
| Liupanshui | 2013 | 1900 | 27593 | 12130 | 1.132855766 | 194 | 0.033 |  |
| Guiyang | 2013 | 8663 | 175858 | 93638 | 0.558248452 | 3508 | 0.032 |  |
| Zunyi | 2013 | 3297 | 68853 | 32554 | 1.50868856 | 2357 | 0.027 |  |
| Chengdu | 2013 | 21152 | 1538274 | 205998 | 0.40052269 | 26114 | 0.031 |  |
| Yibin | 2013 | 3000 | 91300 | 30334 | 1.258683692 | 443 | 0.026 |  |
| Luzhou | 2013 | 2700 | 44001 | 24127 | 1.368980032 | 715 | 0.032 |  |
| Chongqing | 2013 | 52612 | 1764911 | 386529 | 0.573128519 | 23000 | 0.027 |  |
| Yichang | 2013 | 7528 | 302375 | 62912 | 0.435988347 | 3545 | 0.03 |  |
| Jingzhou | 2013 | 3600 | 108760 | 30527 | 0.257448413 | 499 | 0.031 |  |
| Changde | 2013 | 7500 | 215366 | 20342 | 0.823295389 | 783 | 0.02 |  |
| Changsha | 2013 | 35954 | 1094428 | 202244 | 0.284559095 | 5594 | 0.026 |  |
| Yueyang | 2013 | 10795 | 391313.72 | 32452 | 0.706120497 | 710 | 0.022 |  |
| Xianning | 2013 | 1094 | 28956.6 | 20781 | 1.058199321 | 483 | 0.028 |  |
| Wuhan | 2013 | 49323 | 1496423 | 315967 | 0.278912854 | 9779 | 0.024 |  |
| Ezhou | 2013 | 1400 | 63569 | 18251 | 0.534434019 | 1221 | 0.027 |  |
| Huanggang | 2013 | 2300 | 87951 | 49370 | 1.728798657 | 1000 | 0.03 |  |
| Huangshi | 2013 | 3256 | 63350 | 37197 | 0.70534338 | 1164 | 0.025 |  |
| Nanchang | 2013 | 14805 | 535400 | 54172 | 0.469557325 | 2810 | 0.023 |  |
| Jiujiang | 2013 | 872 | 45967 | 38203 | 0.918281 | 430 | 0.025 |  |
| Anqing | 2013 | 2676 | 32838 | 55834 | 1.34621544 | 2915 | 0.026 |  |
| Chizhou | 2013 | 2000 | 302430 | 67887 | 1.075706235 | 962 | 0.024 |  |
| Tongling | 2013 | 1700 | 170700 | 48551 | 0.334952878 | 1054 | 0.019 |  |
| Hefei | 2013 | 24264 | 759825.3 | 260165 | 0.46759225 | 7938 | 0.027 |  |
| Wuhu | 2013 | 5600 | 457575 | 263329 | 0.562469318 | 9763 | 0.025 |  |
| Maanshan | 2013 | 5467 | 236691 | 60893 | 0.542550146 | 3321 | 0.018 |  |
| Nanjing | 2013 | 55537 | 1286467 | 401226 | 0.246503198 | 35610 | 0.027 |  |
| Zhenjiang | 2013 | 18810 | 624375 | 107034 | 0.285144503 | 18447 | 0.021 |  |
| Yangzhou | 2013 | 16460 | 665546 | 104640 | 0.434245158 | 11409 | 0.022 |  |
| Taizhou | 2013 | 17869 | 648021 | 77095 | 0.518623371 | 28000 | 0.019 |  |
| Changzhou | 2013 | 43305 | 1073974 | 221119 | 0.257669279 | 23498 | 0.022 |  |
| Wuxi | 2013 | 71334 | 2117932 | 351021 | 0.179689449 | 40443 | 0.021 |  |
| Nantong | 2013 | 39101 | 1183057 | 190657 | 0.467150653 | 18685 | 0.022 |  |
| Hangzhou | 2013 | 81617 | 2487300 | 462600 | 0.260078334 | 16761 | 0.025 |  |
| Shaoxing | 2013 | 22564 | 828100 | 158729 | 0.341911265 | 12528 | 0.02 |  |
| Huzhou | 2013 | 12553 | 405300 | 60105 | 0.447054735 | 6271 | 0.021 |  |
| Jiaxing | 2013 | 23959 | 769523 | 120567 | 0.337475358 | 4034 | 0.017 |  |
| Suzhou | 2013 | 100181 | 2342437 | 763672 | 0.154261377 | 59000 | 0.021 |  |
| Shanghai | 2013 | 165800 | 7767800 | 2576603 | 0.525211877 | 37770 | 0.023 |  |
| Ningbo | 2013 | 33972 | 1573300 | 375758 | 0.249897642 | 9980 | 0.022 |  |
| Zhoushan | 2013 | 4399 | 126946 | 40225 | 0.363770555 | 827 | 0.021 |  |
| Lijiang | 2014 | 2013 | 19076 | 17960 | 1.519744041 | 168 | 0.016 |  |
| Kunming | 2014 | 5454 | 224528.5 | 125716 | 0.556956039 | 2618 | 0.031 |  |
| Liupanshui | 2014 | 2548 | 31278 | 13476 | 0.982636253 | 179 | 0.013 |  |
| Guiyang | 2014 | 7466 | 177518 | 123754 | 0.47854759 | 8864 | 0.027 |  |
| Zunyi | 2014 | 3764 | 121822 | 35480 | 1.310435028 | 1659 | 0.02 |  |
| Chengdu | 2014 | 24075 | 1538275 | 253590 | 0.375745512 | 33040 | 0.013 |  |
| Yibin | 2014 | 2907 | 97934 | 30468 | 1.198196787 | 1005 | 0.018 |  |
| Luzhou | 2014 | 3270 | 52207 | 25454 | 1.260806294 | 556 | 0.018 |  |
| Chongqing | 2014 | 58354 | 2018528 | 381647 | 0.53945003 | 31000 | 0.018 |  |
| Yichang | 2014 | 8010 | 350856 | 67086 | 0.398966991 | 4921 | 0.022 |  |
| Jingzhou | 2014 | 4298 | 132980 | 38787 | 1.388069119 | 585 | 0.021 |  |
| Changde | 2014 | 8100 | 213100 | 22884 | 0.755622894 | 886 | 0.019 |  |
| Changsha | 2014 | 34328 | 1255732 | 224570 | 0.267798363 | 6315 | 0.027 |  |
| Yueyang | 2014 | 12091 | 435570.6 | 35361 | 0.658612374 | 518 | 0.012 |  |
| Xianning | 2014 | 1093 | 34626 | 24583 | 0.959555779 | 699 | 0.013 |  |
| Wuhan | 2014 | 46992 | 1651122 | 568956 | 0.320422683 | 9931 | 0.019 |  |
| Ezhou | 2014 | 1402 | 76525 | 25864 | 0.500803594 | 1823 | 0.02 |  |
| Huanggang | 2014 | 3048 | 100231 | 55151 | 1.585856855 | 1550 | 0.013 |  |
| Huangshi | 2014 | 3100 | 95000 | 49878 | 0.679081818 | 1420 | 0.022 |  |
| Nanchang | 2014 | 17696 | 594100 | 79045 | 0.440526556 | 3040 | 0.025 |  |
| Jiujiang | 2014 | 1349 | 63355 | 47498 | 0.115986162 | 1088 | 0.021 |  |
| Anqing | 2014 | 2968 | 51755.5 | 61902 | 1.254812904 | 3025 | 0.013 |  |
| Chizhou | 2014 | 2299 | 360160 | 67075 | 0.969185315 | 1619 | 0.018 |  |
| Tongling | 2014 | 2237 | 230700 | 73924 | 0.321550446 | 1108 | 0.011 |  |
| Hefei | 2014 | 28645 | 886822 | 290529 | 0.429428481 | 12671 | 0.02 |  |
| Wuhu | 2014 | 5868 | 528810 | 342871 | 0.519592615 | 8132 | 0.019 |  |
| Maanshan | 2014 | 5798 | 269940.5 | 57483 | 0.531903505 | 3231 | 0.016 |  |
| Nanjing | 2014 | 58591 | 1416149 | 447152 | 0.229533217 | 33264 | 0.026 |  |
| Zhenjiang | 2014 | 23155 | 720875 | 118714 | 0.261103694 | 12472 | 0.02 |  |
| Yangzhou | 2014 | 19818 | 745896 | 113031 | 0.389366464 | 10866 | 0.021 |  |
| Taizhou | 2014 | 23069 | 728663 | 76515 | 0.470804001 | 29118 | 0.021 |  |
| Changzhou | 2014 | 45264 | 1234838 | 217044 | 0.234686069 | 19681 | 0.022 |  |
| Wuxi | 2014 | 68197 | 2136139 | 354743 | 0.181471401 | 26582 | 0.022 |  |
| Nantong | 2014 | 41448 | 1349998 | 217879 | 0.423812309 | 15301 | 0.021 |  |
| Hangzhou | 2014 | 90193 | 2740000 | 524082 | 0.242664803 | 15021 | 0.02 |  |
| Shaoxing | 2014 | 25399 | 922300 | 184555 | 0.324107039 | 10817 | 0.021 |  |
| Huzhou | 2014 | 14057 | 482200 | 69814 | 0.420888554 | 7207 | 0.023 |  |
| Jiaxing | 2014 | 24866 | 867264 | 140870 | 0.324052569 | 4030 | 0.02 |  |
| Suzhou | 2014 | 137151 | 3135422 | 757988 | 0.149938928 | 48540 | 0.021 |  |
| Shanghai | 2014 | 168200 | 8619500 | 2622913 | 0.470331428 | 31176 | 0.027 |  |
| Ningbo | 2014 | 37070 | 1756300 | 428212 | 0.239418184 | 10294 | 0.019 |  |
| Zhoushan | 2014 | 4863 | 139732 | 44763 | 0.343413306 | 1330 | 0.017 |  |
| Lijiang | 2015 | 2511 | 19268 | 19035 | 1.385579381 | 247 | 0.018 |  |
| Kunming | 2015 | 7727 | 320251.4 | 144691 | 0.527529438 | 4265 | 0.024 |  |
| Liupanshui | 2015 | 2097 | 43851 | 33087 | 0.870235783 | 152 | 0.017 |  |
| Guiyang | 2015 | 5719 | 196688 | 145535 | 0.42483351 | 3808 | 0.023 |  |
| Zunyi | 2015 | 3393 | 86795 | 37871 | 1.1470349 | 1210 | 0.018 |  |
| Chengdu | 2015 | 20546 | 1837000 | 390170 | 0.356451744 | 32686 | 0.011 |  |
| Yibin | 2015 | 2613 | 123723 | 31253 | 1.134250035 | 647 | 0.022 |  |
| Luzhou | 2015 | 3021 | 97369 | 35197 | 1.171349713 | 1282 | 0.015 |  |
| Chongqing | 2015 | 61520 | 2470012 | 456689 | 0.513329605 | 43900 | 0.013 |  |
| Yichang | 2015 | 10200 | 577237.1 | 137161 | 0.368794109 | 3298 | 0.015 |  |
| Jingzhou | 2015 | 6217 | 176086 | 52915 | 1.267777837 | 914 | 0.015 |  |
| Changde | 2015 | 9010 | 378858 | 23461 | 0.704970308 | 684 | 0.018 |  |
| Changsha | 2015 | 41019 | 1400388 | 241731 | 0.243807835 | 7366 | 0.011 |  |
| Yueyang | 2015 | 17018 | 468268.7 | 36602 | 0.613026273 | 578 | 0.017 |  |
| Xianning | 2015 | 1343 | 42017 | 30200 | 0.914292572 | 792 | 0.014 |  |
| Wuhan | 2015 | 38040 | 1473266 | 681890 | 0.304936358 | 11880 | 0.014 |  |
| Ezhou | 2015 | 1576 | 107804 | 28081 | 0.473636183 | 1432 | 0.014 |  |
| Huanggang | 2015 | 3551 | 163356 | 68115 | 1.468473421 | 1141 | 0.014 |  |
| Huangshi | 2015 | 3069 | 127630 | 54380 | 0.684053375 | 2300 | 0.016 |  |
| Nanchang | 2015 | 17130 | 637200 | 82004 | 0.407846398 | 3325 | 0.016 |  |
| Jiujiang | 2015 | 1789 | 91670 | 51819 | 0.795136151 | 1192 | 0.019 |  |
| Anqing | 2015 | 2434 | 56381.8 | 71738 | 1.375929323 | 9145 | 0.015 |  |
| Chizhou | 2015 | 2050 | 364107 | 69072 | 0.930079243 | 1980 | 0.014 |  |
| Tongling | 2015 | 2104 | 207500 | 77820 | 0.253803974 | 1628 | 0.012 |  |
| Hefei | 2015 | 30253 | 1040965.5 | 372666 | 0.397516762 | 15294 | 0.016 |  |
| Wuhu | 2015 | 5810 | 852215 | 357319 | 0.490909304 | 11837 | 0.011 |  |
| Maanshan | 2015 | 6866 | 300705.4 | 66451 | 0.524690031 | 3960 | 0.01 |  |
| Nanjing | 2015 | 54850 | 1368297 | 520300 | 0.210724973 | 27995 | 0.02 |  |
| Zhenjiang | 2015 | 26812 | 800583 | 122700 | 0.243167742 | 10767 | 0.015 |  |
| Yangzhou | 2015 | 20528 | 842594 | 131900 | 0.359887673 | 10866 | 0.017 |  |
| Taizhou | 2015 | 25149 | 8311989 | 112100 | 0.431707921 | 23484 | 0.017 |  |
| Changzhou | 2015 | 47431 | 1361163 | 227500 | 0.220479354 | 16974 | 0.016 |  |
| Wuxi | 2015 | 68706 | 2279456 | 359700 | 0.176987283 | 22188 | 0.018 |  |
| Nantong | 2015 | 43364 | 1507497 | 242700 | 0.390982981 | 8800 | 0.018 |  |
| Hangzhou | 2015 | 94323 | 3021900 | 701490 | 0.225715331 | 14594 | 0.018 |  |
| Shaoxing | 2015 | 29718 | 1012000 | 209746 | 0.31104552 | 12029 | 0.012 |  |
| Huzhou | 2015 | 16982 | 532000 | 83942 | 0.39665312 | 5169 | 0.01 |  |
| Jiaxing | 2015 | 30228 | 945131 | 163574 | 0.311467503 | 4748 | 0.01 |  |
| Suzhou | 2015 | 138976 | 3368277 | 883300 | 0.144172213 | 36441 | 0.016 |  |
| Shanghai | 2015 | 171800 | 9361400 | 2718505 | 0.453259405 | 39383 | 0.024 |  |
| Ningbo | 2015 | 42150 | 1931800 | 473092 | 0.229770183 | 12691 | 0.018 |  |
| Zhoushan | 2015 | 4980 | 142682 | 51012 | 0.323941561 | 943 | 0.012 |  |
| Lijiang | 2016 | 2617 | 21445 | 20529 | 1.313293774 | 114 | 0.015 |  |
| Kunming | 2016 | 9459 | 403772 | 151578 | 0.494577261 | 7022 | 0.017 |  |
| Liupanshui | 2016 | 2147 | 48607 | 49294 | 0.817766605 | 201 | 0.018 |  |
| Guiyang | 2016 | 5796 | 229784 | 172621 | 0.401769424 | 5202 | 0.011 |  |
| Zunyi | 2016 | 2970 | 87972 | 61318 | 1.054351304 | 2859 | 0.012 |  |
| Chengdu | 2016 | 17020 | 1840000 | 462043 | 0.3633392 | 56942 | 0.022 |  |
| Yibin | 2016 | 2405 | 158769 | 28069 | 1.063026344 | 990 | 0.013 |  |
| Luzhou | 2016 | 2729 | 144573 | 37880 | 1.084159432 | 1724 | 0.021 |  |
| Chongqing | 2016 | 68055 | 3021830 | 516208 | 0.518810254 | 16800 | 0.018 |  |
| Yichang | 2016 | 11980 | 599929.9 | 136885 | 0.336018588 | 6207 | 0.023 |  |
| Jingzhou | 2016 | 5891 | 200678 | 87332 | 1.183212596 | 1494 | 0.018 |  |
| Changde | 2016 | 11601 | 363320 | 23264 | 0.653869642 | 1471 | 0.021 |  |
| Changsha | 2016 | 37831 | 1369172 | 246112 | 0.23512637 | 14797 | 0.019 |  |
| Yueyang | 2016 | 17978 | 502764 | 54655 | 0.581583991 | 1181 | 0.019 |  |
| Xianning | 2016 | 1389 | 52464 | 37400 | 0.866163016 | 1349 | 0.017 |  |
| Wuhan | 2016 | 35159 | 1283095 | 864219 | 0.285680117 | 21859 | 0.024 |  |
| Ezhou | 2016 | 1670 | 96520 | 29981 | 0.440539959 | 1309 | 0.019 |  |
| Huanggang | 2016 | 4247 | 211596 | 78114 | 1.367684568 | 2345 | 0.014 |  |
| Huangshi | 2016 | 3968 | 200600 | 60684 | 0.653482364 | 2255 | 0.021 |  |
| Nanchang | 2016 | 16030 | 714200 | 101403 | 0.379458544 | 7592 | 0.021 |  |
| Jiujiang | 2016 | 3290 | 115569 | 58853 | 0.731024757 | 2003 | 0.021 |  |
| Anqing | 2016 | 2507 | 60172 | 78775 | 0.952112413 | 13310 | 0.018 |  |
| Chizhou | 2016 | 2124 | 351834 | 67851 | 0.774392397 | 2793 | 0.017 |  |
| Tongling | 2016 | 2093 | 210300 | 83119 | 0.528649431 | 2149 | 0.011 |  |
| Hefei | 2016 | 30576 | 1215243.8 | 1016998 | 0.367684692 | 32296 | 0.026 |  |
| Wuhu | 2016 | 6161 | 739467 | 517841 | 0.429751224 | 16628 | 0.02 |  |
| Maanshan | 2016 | 7615 | 340947.6 | 103014 | 0.48163211 | 4702 | 0.019 |  |
| Nanjing | 2016 | 53267 | 1429259 | 531300 | 0.199473979 | 36416 | 0.027 |  |
| Zhenjiang | 2016 | 28380 | 901853 | 134800 | 0.224247433 | 20424 | 0.022 |  |
| Yangzhou | 2016 | 21960 | 946895 | 122800 | 0.327987016 | 13790 | 0.024 |  |
| Taizhou | 2016 | 27642 | 967142 | 116500 | 0.391647482 | 22489 | 0.021 |  |
| Changzhou | 2016 | 48307 | 1496552 | 240200 | 0.20524552 | 26070 | 0.025 |  |
| Wuxi | 2016 | 73554 | 2425291 | 372400 | 0.166870235 | 41808 | 0.023 |  |
| Nantong | 2016 | 45184 | 1629048.1 | 228329 | 0.358058606 | 21220 | 0.023 |  |
| Hangzhou | 2016 | 94696 | 3463600 | 749190 | 0.205634886 | 32494 | 0.026 |  |
| Shaoxing | 2016 | 32936 | 1128900 | 228725 | 0.293412071 | 42572 | 0.019 |  |
| Huzhou | 2016 | 18963 | 591800 | 93215 | 0.366472009 | 9576 | 0.016 |  |
| Jiaxing | 2016 | 31930 | 1044441 | 179357 | 0.288197696 | 4118 | 0.018 |  |
| Suzhou | 2016 | 148265 | 3615017 | 952000 | 0.138531679 | 5000 | 0.027 |  |
| Shanghai | 2016 | 183900 | 10493200 | 3417109 | 0.415647662 | 55707 | 0.032 |  |
| Ningbo | 2016 | 40779 | 2068100 | 566227 | 0.215049384 | 27272 | 0.021 |  |
| Zhoushan | 2016 | 5526 | 176676.5 | 58786 | 0.285866705 | 1708 | 0.018 |  |
| Lijiang | 2017 | 2784 | 32213 | 20116 | 1.169234365 | 201 | 0.002 |  |
| Kunming | 2017 | 14854 | 534671 | 168930 | 0.451721515 | 8708 | 0.005 |  |
| Liupanshui | 2017 | 2349 | 52187 | 61466 | 0.75591675 | 144 | 0.016 |  |
| Guiyang | 2017 | 6705 | 277373 | 166542 | 0.373348 | 8477 | 0.01 |  |
| Zunyi | 2017 | 4158 | 112432 | 77914 | 0.947634354 | 4164 | 0.015 |  |
| Chengdu | 2017 | 17630 | 2140300 | 532565 | 0.32582695 | 72868 | 0.02 |  |
| Yibin | 2017 | 2077 | 217973 | 29251 | 0.972610106 | 1539 | 0.012 |  |
| Luzhou | 2017 | 2015 | 15771 | 31324 | 1.032759274 | 1766 | 0.018 |  |
| Chongqing | 2017 | 79149 | 3646309 | 593077 | 0.433272462 | 29868 | 0.01 |  |
| Yichang | 2017 | 10105 | 471006 | 143600 | 0.328985671 | 7078 | 0.011 |  |
| Jingzhou | 2017 | 6397 | 246800 | 96425 | 1.080496362 | 2373 | 0.019 |  |
| Changde | 2017 | 10617 | 378858 | 54747 | 0.605277282 | 1965 | 0.017 |  |
| Changsha | 2017 | 40329 | 1712328 | 293220 | 0.217639988 | 19880 | 0.013 |  |
| Yueyang | 2017 | 13265 | 556479.8 | 54559 | 0.563233038 | 1019 | 0.013 |  |
| Xianning | 2017 | 1296 | 56822 | 38180 | 0.796416834 | 1921 | 0.015 |  |
| Wuhan | 2017 | 40179 | 1493485 | 1129006 | 0.205927907 | 24198 | 0.019 |  |
| Ezhou | 2017 | 1769 | 107804 | 43349 | 0.395568667 | 1093 | 0.011 |  |
| Huanggang | 2017 | 4921 | 163356 | 83742 | 1.246150926 | 2292 | 0.009 |  |
| Huangshi | 2017 | 4011 | 143846 | 61241 | 0.592396419 | 3590 | 0.015 |  |
| Nanchang | 2017 | 14606 | 810200 | 217331 | 0.339241366 | 10183 | 0.021 |  |
| Jiujiang | 2017 | 4915 | 168603 | 75892 | 0.697146166 | 3194 | 0.023 |  |
| Anqing | 2017 | 2575 | 70541 | 95817 | 1.004299722 | 5664 | 0.018 |  |
| Chizhou | 2017 | 2237 | 364107 | 62792 | 0.841199993 | 2724 | 0.013 |  |
| Tongling | 2017 | 2381 | 278170 | 83439 | 0.493265569 | 1681 | 0.009 |  |
| Hefei | 2017 | 31512 | 1370590 | 695921 | 0.343112423 | 39871 | 0.014 |  |
| Wuhu | 2017 | 6327 | 852215 | 574155 | 0.423199672 | 20001 | 0.013 |  |
| Maanshan | 2017 | 7925 | 398485 | 123050 | 0.426877094 | 6067 | 0.012 |  |
| Nanjing | 2017 | 51750 | 1532035 | 672942 | 0.187958744 | 43333 | 0.019 |  |
| Zhenjiang | 2017 | 21096 | 904562 | 126531 | 0.213470001 | 18714 | 0.02 |  |
| Yangzhou | 2017 | 22300 | 1032680 | 149805 | 0.293792523 | 18424 | 0.017 |  |
| Taizhou | 2017 | 30711 | 1119764 | 132911 | 0.344460717 | 21552 | 0.019 |  |
| Changzhou | 2017 | 49676 | 1691930 | 250894 | 0.185174499 | 17550 | 0.019 |  |
| Wuxi | 2017 | 82044 | 2680080 | 422110 | 0.15173525 | 23326 | 0.019 |  |
| Nantong | 2017 | 39816 | 1850621 | 272407 | 0.319739719 | 35685 | 0.017 |  |
| Hangzhou | 2017 | 103245 | 3968200 | 923236 | 0.193504097 | 33482 | 0.025 |  |
| Shaoxing | 2017 | 36724 | 1194690 | 260669 | 0.284414836 | 25366 | 0.018 |  |
| Huzhou | 2017 | 19257 | 658970 | 112968 | 0.347710959 | 16783 | 0.018 |  |
| Jiaxing | 2017 | 35976 | 1205487 | 202514 | 0.263179138 | 14785 | 0.022 |  |
| Suzhou | 2017 | 148922 | 3934291 | 1240275 | 0.129080189 | 60471 | 0.017 |  |
| Shanghai | 2017 | 183500 | 12052100 | 3898971 | 0.387130345 | 58934 | 0.017 |  |
| Ningbo | 2017 | 45304 | 2419100 | 587880 | 0.196205916 | 25111 | 0.018 |  |
| Zhoushan | 2017 | 4687 | 125598 | 55548 | 0.257654259 | 1729 | 0.017 |  |

| City | Year | "Transformative" unemployment rate | Number of invention patents granted | Revenue from new product sales | Comprehensive utilization rate of fixed waste | Rentralized treatment rate of wastewater | Excellent air quality rate | Development level of high-tech industries |
| --- | --- | --- | --- | --- | --- | --- | --- | --- |
| Lijiang | 2013 | 0.0354 | 10 | 65327 | 0.9170 | 0.9168 | 1.0000 | 65327 |
| Kunming | 2013 | 0.0267 | 1438 | 2330246 | 0.4090 | 0.9235 | 0.9123 | 2497892.8 |
| Liupanshui | 2013 | 0.039 | 10 | 217758 | 0.4308 | 0.7400 | 0.9350 | 217758 |
| Guiyang | 2013 | 0.0296 | 535 | 3302253 | 0.6075 | 0.9500 | 0.7620 | 241475 |
| Zunyi | 2013 | 0.0291 | 195 | 358330 | 0.9880 | 0.8257 | 0.9450 | 358330 |
| Chengdu | 2013 | 0.0282 | 3196 | 23469703 | 0.9900 | 0.8775 | 0.6080 | 17677589 |
| Yibin | 2013 | 0.0368 | 72 | 3002300 | 0.9109 | 0.9036 | 0.8520 | 1170000 |
| Luzhou | 2013 | 0.0313 | 94 | 1553245 | 0.9000 | 0.4960 | 0.8230 | 1553245 |
| Chongqing | 2013 | 0.034 | 2360 | 26961130 | 0.8400 | 0.9320 | 0.5644 | 25145180 |
| Yichang | 2013 | 0.03 | 243 | 3450200 | 0.4790 | 0.9100 | 0.5490 | 3450200 |
| Jingzhou | 2013 | 0.041 | 80 | 1076290 | 0.3464 | 0.6752 | 0.5010 | 1076300 |
| Changde | 2013 | 0.0395 | 94 | 1403200 | 0.9796 | 0.8581 | 0.7070 | 1538236.5 |
| Changsha | 2013 | 0.0289 | 4027 | 18831765 | 0.8567 | 0.9632 | 0.6410 | 14992400.2 |
| Yueyang | 2013 | 0.0396 | 113 | 3905421 | 0.9300 | 0.8895 | 0.8790 | 4056877.7 |
| Xianning | 2013 | 0.0288 | 29 | 417537 | 0.5620 | 0.9100 | 0.9450 | 466100 |
| Wuhan | 2013 | 0.0352 | 3171 | 17508626 | 0.9500 | 0.9250 | 0.5025 | 17001874 |
| Ezhou | 2013 | 0.0332 | 40 | 902000 | 0.9022 | 0.8870 | 0.8027 | 902000 |
| Huanggang | 2013 | 0.04 | 46 | 967700 | 0.9233 | 0.5687 | 0.8220 | 967700 |
| Huangshi | 2013 | 0.0218 | 61 | 1771300 | 0.9431 | 0.9040 | 0.8658 | 1771300 |
| Nanchang | 2013 | 0.0315 | 897 | 5019100 | 0.9780 | 0.9420 | 0.6082 | 5019100 |
| Jiujiang | 2013 | 0.045 | 89 | 1668245 | 0.4740 | 0.8784 | 0.9370 | 1860900 |
| Anqing | 2013 | 0.0411 | 78 | 1737050 | 0.9690 | 0.8650 | 0.7918 | 1737050 |
| Chizhou | 2013 | 0.032 | 113 | 1798370 | 1.0000 | 0.9107 | 0.9620 | 179837.98 |
| Tongling | 2013 | 0.0316 | 138 | 1726000 | 0.8312 | 0.8569 | 0.8904 | 3865093 |
| Hefei | 2013 | 0.0325 | 1547 | 13624549 | 0.9327 | 0.8710 | 0.4990 | 13624550 |
| Wuhu | 2013 | 0.035 | 750 | 6847260 | 0.9810 | 0.9260 | 0.8600 | 8437263 |
| Maanshan | 2013 | 0.028 | 280 | 2533014 | 0.7021 | 0.8700 | 0.7180 | 3178002 |
| Nanjing | 2013 | 0.0215 | 4729 | 20998489 | 0.9120 | 0.6150 | 0.5534 | 6631800 |
| Zhenjiang | 2013 | 0.0212 | 874 | 8517256 | 0.9810 | 0.7860 | 0.5930 | 7461400 |
| Yangzhou | 2013 | 0.0226 | 406 | 9027636 | 0.9770 | 0.8360 | 0.6490 | 5440800 |
| Taizhou | 2013 | 0.0216 | 248 | 8137549 | 0.9820 | 0.6140 | 0.8680 | 11354700 |
| Changzhou | 2013 | 0.0214 | 1173 | 17021259 | 0.9820 | 0.8740 | 0.5863 | 9165100 |
| Wuxi | 2013 | 0.0212 | 2713 | 26439439 | 0.9100 | 0.8660 | 0.8160 | 6810900 |
| Nantong | 2013 | 0.0215 | 746 | 16928113 | 0.9800 | 0.8840 | 0.6140 | 12931200 |
| Hangzhou | 2013 | 0.0185 | 4903 | 31007253 | 0.9400 | 0.9390 | 0.5890 | 11744200 |
| Shaoxing | 2013 | 0.0289 | 729 | 24874400 | 0.9270 | 0.8619 | 0.6820 | 11616000 |
| Huzhou | 2013 | 0.0307 | 506 | 6464000 | 0.9627 | 0.9140 | 0.5210 | 1646400 |
| Jiaxing | 2013 | 0.0293 | 407 | 24675526 | 0.9500 | 0.9100 | 0.8660 | 16237339 |
| Suzhou | 2013 | 0.0212 | 4413 | 68576200 | 0.9790 | 0.7720 | 0.7260 | 68576200 |
| Shanghai | 2013 | 0.04 | 10644 | 76883800 | 0.9712 | 0.8770 | 0.6600 | 16705524 |
| Ningbo | 2013 | 0.0216 | 2246 | 9834700 | 0.9006 | 0.7700 | 0.6590 | 9834700 |
| Zhoushan | 2013 | 0.027 | 211 | 1803124 | 0.9983 | 0.6410 | 0.9010 | 2112533 |
| Lijiang | 2014 | 0.0386 | 10 | 57821 | 0.8900 | 0.8515 | 1.0000 | 57821 |
| Kunming | 2014 | 0.0234 | 1501 | 1788277 | 0.3687 | 0.9015 | 0.9699 | 3612847 |
| Liupanshui | 2014 | 0.039 | 10 | 335459 | 0.5440 | 0.7574 | 0.9210 | 135459 |
| Guiyang | 2014 | 0.0314 | 674 | 3654209 | 0.4886 | 0.9520 | 0.8600 | 318683 |
| Zunyi | 2014 | 0.028 | 216 | 437753 | 0.9433 | 0.8382 | 0.7750 | 437753 |
| Chengdu | 2014 | 0.0287 | 4021 | 25567279 | 0.9744 | 0.8800 | 0.6110 | 13004452 |
| Yibin | 2014 | 0.0376 | 103 | 3564000 | 0.7345 | 0.8125 | 0.8490 | 1230000 |
| Luzhou | 2014 | 0.0314 | 83 | 1075400 | 0.9701 | 0.4969 | 0.7260 | 1075400 |
| Chongqing | 2014 | 0.035 | 2321 | 36107819 | 0.8449 | 0.9225 | 0.6740 | 33885785 |
| Yichang | 2014 | 0.0223 | 277 | 4752900 | 0.6288 | 0.9067 | 0.6210 | 4762900 |
| Jingzhou | 2014 | 0.045 | 89 | 1290067 | 0.3821 | 0.8840 | 0.5213 | 1292100 |
| Changde | 2014 | 0.0416 | 103 | 1764000 | 0.9742 | 0.8609 | 0.6980 | 1763779 |
| Changsha | 2014 | 0.0285 | 4128 | 24885535 | 0.8550 | 0.9980 | 0.6230 | 22319197 |
| Yueyang | 2014 | 0.0402 | 119 | 4649384 | 0.8820 | 0.8827 | 0.7400 | 4649384 |
| Xianning | 2014 | 0.0257 | 33 | 649372 | 0.5471 | 0.9100 | 0.9040 | 639300 |
| Wuhan | 2014 | 0.0315 | 3874 | 16569475 | 0.9871 | 0.9300 | 0.4986 | 19949152 |
| Ezhou | 2014 | 0.0372 | 46 | 1001400 | 0.8875 | 0.8197 | 0.8250 | 1001400 |
| Huanggang | 2014 | 0.0311 | 53 | 1175800 | 0.9144 | 0.8500 | 0.8465 | 1175800 |
| Huangshi | 2014 | 0.0226 | 72 | 1920400 | 0.9342 | 0.9060 | 0.8630 | 1920400 |
| Nanchang | 2014 | 0.035 | 927 | 5333968 | 0.9591 | 0.9100 | 0.8050 | 5333968 |
| Jiujiang | 2014 | 0.045 | 95 | 1313458 | 0.6038 | 0.8661 | 0.8140 | 2426100 |
| Anqing | 2014 | 0.0368 | 77 | 1907132 | 0.9654 | 0.8601 | 0.9068 | 1907132 |
| Chizhou | 2014 | 0.036 | 62 | 1519308 | 0.8522 | 0.9224 | 0.9930 | 519308 |
| Tongling | 2014 | 0.0337 | 145 | 1806000 | 0.8316 | 0.9014 | 0.8164 | 6311595 |
| Hefei | 2014 | 0.0296 | 1891 | 16325355 | 0.9302 | 0.8759 | 0.5280 | 16325356 |
| Wuhu | 2014 | 0.035 | 853 | 9886491 | 0.9332 | 0.8996 | 0.6930 | 9886491 |
| Maanshan | 2014 | 0.028 | 385 | 2338829 | 0.7106 | 0.8783 | 0.8550 | 3014269 |
| Nanjing | 2014 | 0.019 | 5265 | 18626575 | 0.9190 | 0.6540 | 0.5205 | 3382100 |
| Zhenjiang | 2014 | 0.0191 | 1274 | 18392167 | 0.9860 | 0.7990 | 0.6590 | 3402400 |
| Yangzhou | 2014 | 0.0208 | 467 | 11120259 | 0.9230 | 0.8440 | 0.6550 | 2300500 |
| Taizhou | 2014 | 0.0195 | 345 | 12495057 | 0.9830 | 0.6340 | 0.6580 | 1153900 |
| Changzhou | 2014 | 0.019 | 1696 | 20107879 | 0.9820 | 0.8910 | 0.6380 | 3341600 |
| Wuxi | 2014 | 0.0191 | 2801 | 29471732 | 0.9110 | 0.8710 | 0.5770 | 3795300 |
| Nantong | 2014 | 0.019 | 932 | 20477383 | 0.9830 | 0.8620 | 0.7080 | 2881100 |
| Hangzhou | 2014 | 0.0184 | 5552 | 35238845 | 0.9110 | 0.9387 | 0.6250 | 10235676 |
| Shaoxing | 2014 | 0.0276 | 880 | 29699700 | 0.9720 | 0.8789 | 0.7205 | 424800 |
| Huzhou | 2014 | 0.0298 | 700 | 8434400 | 0.9659 | 0.9198 | 0.6080 | 2434400 |
| Jiaxing | 2014 | 0.0292 | 546 | 26565483 | 0.9601 | 0.9037 | 0.7030 | 15887430 |
| Suzhou | 2014 | 0.0192 | 5264 | 63781800 | 0.9670 | 0.7960 | 0.7180 | 53781800 |
| Shanghai | 2014 | 0.042 | 11614 | 84469600 | 0.9751 | 0.8980 | 0.7700 | 23101273 |
| Ningbo | 2014 | 0.0195 | 2832 | 10035278 | 0.9076 | 0.8111 | 0.7210 | 10035278 |
| Zhoushan | 2014 | 0.0271 | 258 | 2035442 | 0.9980 | 0.6326 | 0.9400 | 2130700 |
| Lijiang | 2015 | 0.0387 | 12 | 90001 | 0.8130 | 0.8693 | 1.0000 | 90001 |
| Kunming | 2015 | 0.036151 | 1586 | 2317940.8 | 0.3636 | 0.9197 | 0.9781 | 4079995 |
| Liupanshui | 2015 | 0.0399 | 12 | 378583 | 0.5794 | 0.8850 | 0.9900 | 109440 |
| Guiyang | 2015 | 0.0312 | 1025 | 4756622 | 0.4815 | 0.9850 | 0.9320 | 312245 |
| Zunyi | 2015 | 0.0284 | 298 | 521110 | 0.6300 | 0.8990 | 0.9100 | 521100 |
| Chengdu | 2015 | 0.0319 | 6206 | 20149000 | 0.9606 | 0.9000 | 0.5863 | 10999552 |
| Yibin | 2015 | 0.039 | 178 | 4320000 | 0.9644 | 0.8097 | 0.7230 | 1300000 |
| Luzhou | 2015 | 0.038923 | 100 | 1110153 | 0.9715 | 0.5891 | 0.7500 | 1440000 |
| Chongqing | 2015 | 0.036 | 3964 | 45351174 | 0.8445 | 0.9367 | 0.8000 | 40283440 |
| Yichang | 2015 | 0.018 | 229 | 5923200 | 0.2627 | 0.9168 | 0.6904 | 5923200 |
| Jingzhou | 2015 | 0.045 | 541 | 1540961 | 0.3090 | 0.8853 | 0.6000 | 1546800 |
| Changde | 2015 | 0.0258 | 143 | 2117000 | 0.9526 | 0.9254 | 0.7670 | 2178050 |
| Changsha | 2015 | 0.026 | 4323 | 29954658 | 0.8620 | 0.9980 | 0.7070 | 25598479 |
| Yueyang | 2015 | 0.028 | 176 | 5366363 | 0.8554 | 0.7564 | 0.7460 | 5365595 |
| Xianning | 2015 | 0.014 | 50 | 729747 | 0.0049 | 0.9100 | 0.6770 | 857018 |
| Wuhan | 2015 | 0.0308 | 5999 | 14751598 | 0.9796 | 0.9500 | 0.5260 | 22356480 |
| Ezhou | 2015 | 0.031 | 66 | 1456719 | 0.9816 | 0.8205 | 0.6730 | 1146459 |
| Huanggang | 2015 | 0.031 | 118 | 1300000 | 0.9035 | 0.6529 | 0.6550 | 1304766 |
| Huangshi | 2015 | 0.0234 | 102 | 1560500 | 0.9203 | 0.9170 | 0.6710 | 1560500 |
| Nanchang | 2015 | 0.035 | 932 | 6783852 | 0.9710 | 0.9772 | 0.8630 | 3865337 |
| Jiujiang | 2015 | 0.019505 | 102 | 1641728 | 0.6036 | 0.8801 | 0.8380 | 2558651 |
| Anqing | 2015 | 0.0304 | 309 | 1709000 | 0.9680 | 0.8818 | 0.8400 | 1569862 |
| Chizhou | 2015 | 0.0284 | 203 | 1701900 | 0.9397 | 0.9358 | 0.9450 | 668941 |
| Tongling | 2015 | 0.0351 | 274 | 1679000 | 0.9058 | 0.9207 | 0.7863 | 1679167 |
| Hefei | 2015 | 0.028 | 3413 | 20126687 | 0.9165 | 0.9212 | 0.6630 | 10284711 |
| Wuhu | 2015 | 0.0329 | 1927 | 9503112 | 0.8644 | 0.9079 | 0.7730 | 6271081 |
| Maanshan | 2015 | 0.029 | 930 | 1750685 | 0.8651 | 0.9210 | 0.7530 | 380950 |
| Nanjing | 2015 | 0.019 | 8244 | 18117321 | 0.9050 | 0.6449 | 0.6438 | 54061072 |
| Zhenjiang | 2015 | 0.0189 | 2797 | 20333333 | 0.9518 | 0.8140 | 0.7000 | 4408867 |
| Yangzhou | 2015 | 0.0201 | 754 | 10089737 | 0.9750 | 0.8545 | 0.6790 | 4581334 |
| Taizhou | 2015 | 0.0189 | 651 | 12530640 | 0.9851 | 0.6443 | 0.6820 | 6657264 |
| Changzhou | 2015 | 0.0189 | 2664 | 23147640 | 0.9832 | 0.9039 | 0.6730 | 1727831 |
| Wuxi | 2015 | 0.0189 | 5480 | 28403282 | 0.9440 | 0.8940 | 0.6410 | 1027023 |
| Nantong | 2015 | 0.0185 | 2217 | 22343847 | 0.9887 | 0.9158 | 0.6770 | 8040377 |
| Hangzhou | 2015 | 0.0174 | 8296 | 40250559 | 0.8860 | 0.9428 | 0.6630 | 12130603 |
| Shaoxing | 2015 | 0.0256 | 1523 | 34182600 | 0.9690 | 0.9057 | 0.7390 | 4305195 |
| Huzhou | 2015 | 0.0267 | 1647 | 13241938 | 0.9787 | 0.9250 | 0.5970 | 3241938 |
| Jiaxing | 2015 | 0.0288 | 1184 | 26065636 | 0.9437 | 0.9019 | 0.6440 | 6212452 |
| Suzhou | 2015 | 0.02 | 10488 | 67667872 | 0.9811 | 0.8255 | 0.6690 | 17667872 |
| Shanghai | 2015 | 0.041 | 17601 | 74709300 | 0.9615 | 0.9100 | 0.7070 | 27660974 |
| Ningbo | 2015 | 0.0201 | 5412 | 10620790 | 0.9480 | 0.7944 | 0.8270 | 10620790 |
| Zhoushan | 2015 | 0.016002 | 406 | 2625882 | 0.9960 | 0.6480 | 0.7080 | 2451520 |
| Lijiang | 2016 | 0.0381 | 13 | 141445 | 0.6700 | 0.8861 | 1.0000 | 41445 |
| Kunming | 2016 | 0.0311 | 1637 | 2703581 | 0.4300 | 0.9148 | 0.8890 | 5590101 |
| Liupanshui | 2016 | 0.0397 | 13 | 239196 | 0.6250 | 0.6738 | 0.9640 | 239196 |
| Guiyang | 2016 | 0.0309 | 1072 | 5154733 | 0.3913 | 0.9600 | 0.9560 | 1073621 |
| Zunyi | 2016 | 0.0277 | 333 | 764453 | 0.5280 | 0.9489 | 0.9290 | 764453 |
| Chengdu | 2016 | 0.033 | 7990 | 23660000 | 0.9700 | 0.9500 | 0.5863 | 23013905 |
| Yibin | 2016 | 0.0393 | 190 | 5031900 | 0.8300 | 0.6802 | 0.7280 | 2105986 |
| Luzhou | 2016 | 0.0351 | 94 | 1179600 | 0.9718 | 0.7471 | 0.6400 | 1179600 |
| Chongqing | 2016 | 0.037 | 6138 | 50143454 | 0.7690 | 0.9537 | 0.8246 | 4214112 |
| Yichang | 2016 | 0.018 | 666 | 6662400 | 0.2526 | 0.9313 | 0.6767 | 6763790 |
| Jingzhou | 2016 | 0.0254 | 272 | 1627500 | 0.3944 | 0.8971 | 0.6493 | 1631680 |
| Changde | 2016 | 0.0313 | 158 | 2600000 | 0.9637 | 0.9100 | 0.7300 | 2599362 |
| Changsha | 2016 | 0.0274 | 4656 | 34328182 | 0.9400 | 1.0000 | 0.7300 | 28678929 |
| Yueyang | 2016 | 0.036 | 188 | 6106381 | 0.7198 | 0.7765 | 0.7760 | 6105613 |
| Xianning | 2016 | 0.0178 | 64 | 970056 | 0.9957 | 0.8500 | 0.7560 | 989360 |
| Wuhan | 2016 | 0.0294 | 8444 | 14234302 | 0.9745 | 0.9560 | 0.6493 | 24182598 |
| Ezhou | 2016 | 0.0174 | 41 | 1311058 | 0.8452 | 0.8529 | 0.6420 | 1311058 |
| Huanggang | 2016 | 0.0131 | 122 | 1568100 | 0.5417 | 0.6740 | 0.6960 | 1574826 |
| Huangshi | 2016 | 0.0238 | 144 | 1565700 | 0.9352 | 0.9038 | 0.7315 | 1584696 |
| Nanchang | 2016 | 0.0351 | 1084 | 7793895 | 0.9500 | 0.9800 | 0.8690 | 4707775 |
| Jiujiang | 2016 | 0.0381 | 110 | 2483195 | 0.6410 | 0.9002 | 0.7920 | 3010899 |
| Anqing | 2016 | 0.0322 | 438 | 1365813 | 0.9733 | 0.8838 | 0.9867 | 1365813 |
| Chizhou | 2016 | 0.0329 | 98 | 1769260 | 0.8834 | 0.9384 | 0.7970 | 769260 |
| Tongling | 2016 | 0.0309 | 537 | 1886000 | 0.9237 | 0.9300 | 0.7459 | 1885783 |
| Hefei | 2016 | 0.0303 | 4789 | 25096371 | 0.7365 | 0.9163 | 0.7048 | 12944046 |
| Wuhu | 2016 | 0.0339 | 2917 | 8233292 | 0.9162 | 0.9356 | 0.8030 | 8233292 |
| Maanshan | 2016 | 0.0293 | 1075 | 2215273 | 0.9102 | 0.9466 | 0.7430 | 1275671 |
| Nanjing | 2016 | 0.0188 | 10614 | 20389124 | 0.8580 | 0.6561 | 0.6630 | 59026382 |
| Zhenjiang | 2016 | 0.0185 | 2593 | 22887253 | 0.9100 | 0.8310 | 0.7640 | 1844738 |
| Yangzhou | 2016 | 0.0188 | 768 | 10700347 | 0.9730 | 0.8600 | 0.7160 | 3206644 |
| Taizhou | 2016 | 0.0187 | 944 | 16433143 | 0.9890 | 0.7103 | 0.7400 | 6796024 |
| Changzhou | 2016 | 0.0185 | 2830 | 23751245 | 0.9810 | 0.9230 | 0.6740 | 1845017 |
| Wuxi | 2016 | 0.0185 | 4826 | 28126060 | 0.9490 | 0.9070 | 0.6690 | 1220415 |
| Nantong | 2016 | 0.0185 | 2630 | 22805469 | 0.9560 | 0.8700 | 0.7190 | 7835301 |
| Hangzhou | 2016 | 0.0172 | 9872 | 46423002 | 0.8512 | 0.9446 | 0.7123 | 13735196 |
| Shaoxing | 2016 | 0.0236 | 2118 | 37130400 | 0.9458 | 0.9363 | 0.8030 | 4808187 |
| Huzhou | 2016 | 0.0252 | 2190 | 13474527 | 0.9925 | 0.9271 | 0.6222 | 3474527 |
| Jiaxing | 2016 | 0.0287 | 1850 | 31665233 | 0.9212 | 0.9029 | 0.7430 | 7137179 |
| Suzhou | 2016 | 0.0189 | 11618 | 69480993 | 0.8880 | 0.8353 | 0.6900 | 19480993 |
| Shanghai | 2016 | 0.041 | 20681 | 90334800 | 0.9570 | 0.9300 | 0.7540 | 41748624 |
| Ningbo | 2016 | 0.0201 | 5382 | 11535659 | 0.9486 | 0.8442 | 0.8470 | 11535659 |
| Zhoushan | 2016 | 0.0298 | 501 | 3227501 | 0.9270 | 0.7162 | 0.8420 | 2254446 |
| Lijiang | 2017 | 0.0351 | 14 | 146169 | 0.4329 | 0.9335 | 1.0000 | 46169 |
| Kunming | 2017 | 0.03 | 1756 | 3276324 | 0.5400 | 0.9237 | 0.9860 | 6489807 |
| Liupanshui | 2017 | 0.0383 | 14 | 378583 | 0.5774 | 0.8700 | 0.9400 | 378583 |
| Guiyang | 2017 | 0.0313 | 1072 | 5752405 | 0.3386 | 0.9708 | 0.9510 | 1326735 |
| Zunyi | 2017 | 0.029 | 349 | 1246085 | 0.6263 | 0.8370 | 0.9420 | 3246085 |
| Chengdu | 2017 | 0.032 | 7990 | 25703500 | 0.9600 | 0.9400 | 0.6438 | 16653379 |
| Yibin | 2017 | 0.0395 | 229 | 5853600 | 0.7154 | 0.8500 | 0.7151 | 2464205 |
| Luzhou | 2017 | 0.0323 | 94 | 1810153 | 0.9720 | 0.7748 | 0.9196 | 3810153 |
| Chongqing | 2017 | 0.034 | 5044 | 53227016 | 0.7000 | 0.9371 | 0.8301 | 15990246 |
| Yichang | 2017 | 0.0206 | 666 | 4064100 | 0.2511 | 0.9349 | 0.7068 | 4065457 |
| Jingzhou | 2017 | 0.0215 | 243 | 1910400 | 0.2827 | 0.9181 | 0.8316 | 1628086 |
| Changde | 2017 | 0.031 | 164 | 3258800 | 0.9857 | 0.9674 | 0.7530 | 3691434 |
| Changsha | 2017 | 0.0267 | 4873 | 37301627 | 0.8242 | 0.9612 | 0.7898 | 35104319 |
| Yueyang | 2017 | 0.0348 | 215 | 6172346 | 0.7600 | 0.8799 | 0.8360 | 6444383 |
| Xianning | 2017 | 0.0214 | 57 | 1581325 | 0.8559 | 0.8808 | 0.7950 | 1247209 |
| Wuhan | 2017 | 0.0284 | 8444 | 18795686 | 0.9704 | 0.9600 | 0.6986 | 26699987 |
| Ezhou | 2017 | 0.0203 | 156 | 1456719 | 0.8015 | 0.9400 | 0.7470 | 1456719 |
| Huanggang | 2017 | 0.0191 | 97 | 1954200 | 0.8563 | 0.6808 | 0.7420 | 1966032 |
| Huangshi | 2017 | 0.0283 | 144 | 1958400 | 0.9346 | 0.8996 | 0.8258 | 1952808 |
| Nanchang | 2017 | 0.0338 | 1084 | 8728450 | 0.9600 | 0.9970 | 0.8300 | 6719284 |
| Jiujiang | 2017 | 0.0342 | 114 | 2567279 | 0.5380 | 0.8885 | 0.7890 | 3650728 |
| Anqing | 2017 | 0.0262 | 478 | 1596000 | 0.9461 | 0.8981 | 0.7342 | 1855000 |
| Chizhou | 2017 | 0.0314 | 98 | 1901419 | 0.9574 | 0.9474 | 0.6880 | 901419 |
| Tongling | 2017 | 0.0286 | 213 | 1485124 | 0.9181 | 0.9302 | 0.7123 | 1485124 |
| Hefei | 2017 | 0.0286 | 4799 | 27350435 | 0.8426 | 0.9417 | 0.6751 | 15242020 |
| Wuhu | 2017 | 0.0305 | 2971 | 9302300 | 0.7994 | 0.9251 | 0.6880 | 9503112 |
| Maanshan | 2017 | 0.0298 | 1181 | 2679800 | 0.9256 | 0.9321 | 0.6560 | 1167538 |
| Nanjing | 2017 | 0.0182 | 10723 | 22470780 | 0.9040 | 0.7100 | 0.7233 | 11715100 |
| Zhenjiang | 2017 | 0.0182 | 2693 | 14840262 | 0.9722 | 0.8574 | 0.7200 | 36445715 |
| Yangzhou | 2017 | 0.0184 | 738 | 11230568 | 0.9700 | 0.8600 | 0.6250 | 4522974 |
| Taizhou | 2017 | 0.0182 | 956 | 15982823 | 0.9900 | 0.7947 | 0.7420 | 763869 |
| Changzhou | 2017 | 0.018 | 2830 | 24577963 | 0.9960 | 0.9310 | 0.6820 | 4482186 |
| Wuxi | 2017 | 0.0182 | 4825 | 33950311 | 0.9100 | 0.9300 | 0.6770 | 1676300 |
| Nantong | 2017 | 0.024 | 2630 | 21585268 | 0.9600 | 0.8775 | 0.7290 | 4914425 |
| Hangzhou | 2017 | 0.017 | 8647 | 42120141 | 0.7706 | 0.9510 | 0.7425 | 16119697 |
| Shaoxing | 2017 | 0.0225 | 1754 | 29170000 | 0.9111 | 0.9515 | 0.8300 | 5713166 |
| Huzhou | 2017 | 0.0222 | 2544 | 15713166 | 0.9925 | 0.9496 | 0.7535 | 3216493 |
| Jiaxing | 2017 | 0.0282 | 1654 | 33763880 | 0.9794 | 0.9269 | 0.7260 | 8182811 |
| Suzhou | 2017 | 0.0182 | 11618 | 74548388 | 0.9340 | 0.8900 | 0.7150 | 14548388 |
| Shanghai | 2017 | 0.039 | 20086 | 100681500 | 0.9400 | 0.9450 | 0.7530 | 13653670 |
| Ningbo | 2017 | 0.02 | 5669 | 20944135 | 0.9555 | 0.8295 | 0.8520 | 20964135 |
| Zhoushan | 2017 | 0.0282 | 497 | 2159294 | 0.9500 | 0.8305 | 0.7311 | 459857 |
